# Multi-omics insights into microbiome-rumen epithelium interaction mechanisms underlying subacute rumen acidosis tolerance in dairy goats

**DOI:** 10.1186/s13059-025-03789-y

**Published:** 2025-10-09

**Authors:** Jingyi Xu, Xiaodong Chen, Jianrong Ren, Jiawen Xu, Lei Zhang, Fang Yan, Tao Liu, Guijie Zhang, Sharon A. Huws, Junhu Yao, Shengru Wu

**Affiliations:** 1 College of Animal Science and Technology, Northwest A&F University, Yangling , Shaanxi, 712100 China; 2 College of Animal Science and Technology, Ningxia University, Yinchuan, 750021 China; 3 Key Laboratory of Livestock Biology, Northwest A&F University, Yangling , Shaanxi, 712100 China; 4 Institute of Global Food Security, School of Biological Sciences, Queen’s University Belfast, 19 Chlorine Gardens, Belfast, Northern Ireland BT9 5DL UK; 5 National Center of Technology Innovation for Dairy, Nei Mongol Autonomous Region, Hohhot, 010010 China

**Keywords:** Dairy goats, Subacute rumen acidosis, Rumen microbiome and metabolites, Single-nucleus transcriptome

## Abstract

**Background:**

To address rising demand for dairy products, dairy goats are often fed high-concentrate diets, which lead to subacute rumen acidosis (SARA). The mechanisms behind individual variation in SARA tolerance are not well understood. This study aims to elucidate roles of rumen microbiome-host interactions in SARA-susceptibility and tolerance.

**Results:**

Goats susceptible or tolerant to SARA were selected by feeding diets with different levels of rumen degradable starch. SARA-susceptible goats present prolonged periods of rumen pH below 5.8 and volatile fatty acids (VFAs) accumulation. Metagenomic analysis reveals a decrease in cellulose- and hemicellulose-utilizing bacteria and enzymes, along with increased lysozymes, suggesting disrupted rumen homeostasis. Transcriptomic and single-nucleus transcriptome analyses reveal upregulated Th17 cells, IL-17 signalling, and inflammatory pathways in SARA-susceptible goats. In contrast, SARA-tolerant goats maintain stable pH levels and enhance VFAs absorption. *Bifidobacterium adolescentis* and other beneficial bacteria are enriched in the rumen of SARA-tolerant goats. These microbes are positively correlated with 3-methyl pyruvic acid, a key metabolite involved in branched-chain amino acid synthesis and epithelial cell proliferation. Both microbiome transplantation and *B. adolescentis* direct feeding experiments confirm the protective effects of SARA-tolerant microbiota including *B. adolescentis*, promoting rumen epithelial VFAs absorption and reducing ruminal inflammation.

**Conclusions:**

This study highlights the importance of Th17-mediated immune responses in ruminal inflammation and the role of *B. adolescentis* in regulating rumen epithelial VFAs absorption. Modulating VFAs absorption in the rumen epithelium represents a promising strategy for improving animal health and enhancing rumen fermentation efficiency.

**Supplementary Information:**

The online version contains supplementary material available at 10.1186/s13059-025-03789-y.

## Background

Food security has never been so crucial given that the world population is growing and 11% of the human population is currently malnutritioned. Ruminant products offer nutrient-dense food; however, demand is likely to outcompete resources very soon. In particular, goat milk consumption is increasing, due to its higher medium- and short-chain fatty acids, vitamin, β-casein, and trace minerals, and decreased α-casein and allergens compared to cow’s milk [1, 2]. As such, sustainably increasing high-quality goat milk yield has become one of the most important outcomes for dairy goats.

To more effectively meet the nutritional needs of dairy goats and ensure high milk production, the general practice is to feed lactating goats high-concentrate diets [3–7]. As a consequence, these animals often suffer from subacute ruminal acidosis (SARA) [8–10], which leads to a reduced ruminal pH, decreased fiber degradation capacity and VFAs production, and low feed intake, which results in poor health and decreased productivity [10–13]. Past researches have suggested that the ruminal microbiota, including the increased abundance of starch-degrading microorganisms, contributes to the occurrence of SARA [8, 13–15]. Hence, it has been suggested that SARA could be avoided in dairy goats fed with high-starch diet through regulation of the rumen microbiome. However, not all ruminants fed a high-concentrate diet develop SARA with some being very susceptible [14, 16, 17] and others being tolerant [18–20]. The mechanisms underlying this difference in susceptibility in individual ruminants are unknown. The interplay between microbes and their host holds significant importance in metabolic alterations and disease prevention, garnering extensive researches attention in recent years [21–23]. In that case, identifying the ruminal feature microorganisms of the SARA tolerance goats can help study the potential microbial mechanism to prevent SARA [15, 24, 25].

Along with a decrease in ruminal pH, the lysis of some Gram-negative bacteria can lead to the substantial release of lipopolysaccharide, which can activate the TLR/MyD88-NFκB pathway and trigger an inflammatory response in the rumen epithelium of dairy goats [26–28]. Gastrointestinal inflammation is regulated by a complex immune network, and the nonspecific innate immune processes mediated by lipopolysaccharide stimulation may not fully account for the mechanisms underlying the inflammatory response in dairy goats with SARA. During the inflammatory process in SARA-affected dairy goats, there may be multiple triggers involving immune cells [14, 19]. For instance, studies have shown that the regulation of mammalian intestinal inflammation is primarily mediated by T helper cells [29–31]. Therefore, elucidating the changes in rumen microbiome-induced alterations in rumen immune cells in SARA-affected dairy goats contributes to a deeper understanding of the pathogenesis of SARA. Furthermore, when ruminants are fed high-concentrate diets, those which are SARA tolerant can maintain the ruminal inter-environment stable, avoid the ruminal inflammation and the occurrence of SARA [14, 18, 19, 32]. Hence, identifying the ruminal epithelial cell and immune cell compositions and gene expression features of these individuals and further studying their interactions with the ruminal microbiome can help better understand the host-microbe interaction mechanisms underlying the differences in susceptibility of individual dairy ruminants to SARA.

As such, we hypothesize that the variations in rumen microbiome and its metabolites are responsible for the different susceptibilities to SARA through affecting the ruminal cell subtype composition and their transcriptomic dynamics in the rumen. Recently, the application of omics approaches, especially the integrated meta-omics based on the metagenome or single-nucleus RNA sequencing (snRNA-seq), has provided more knowledge related to the host-microbiome interactions in ruminants [33–36]. Therefore, in this study, we aimed to use these novel multiomic technologies to address the following research questions: (1) How the rumen cell subtypes, especially the ruminal immune cell composition and gene expression change during when SARA occurrence? (2) Are individualized ruminal microbiome involved in the regulation of varied SARA responses? (3) What are the interactions among the ruminal microbiome, rumen epithelial cells, and immune cells and do these interactions coregulate SARA tolerance when goats are fed with the high-concentrate feed?

Here, we explored the discrepancies of rumen microbiome, metabolites, rumen epithelial gene expression, and cell subtypes, which discovered for the first time that the high abundance of *Bifidobacterium adolescentis* in SARA-tolerant dairy goats can maintain the stability of rumen, promote the proliferation of rumen epithelial cells, and enhance the absorption of VFAs by the rumen epithelium, thereby leading to SARA tolerance. Furthermore, the results of the rumen microbiome transplantation and *Bifidobacterium adolescentis* feeding experiments corroborated our findings.

## Results

### Rumen fermentation was altered and epithelial injury occurred under the high RDS feeding

**Fig. 1:**
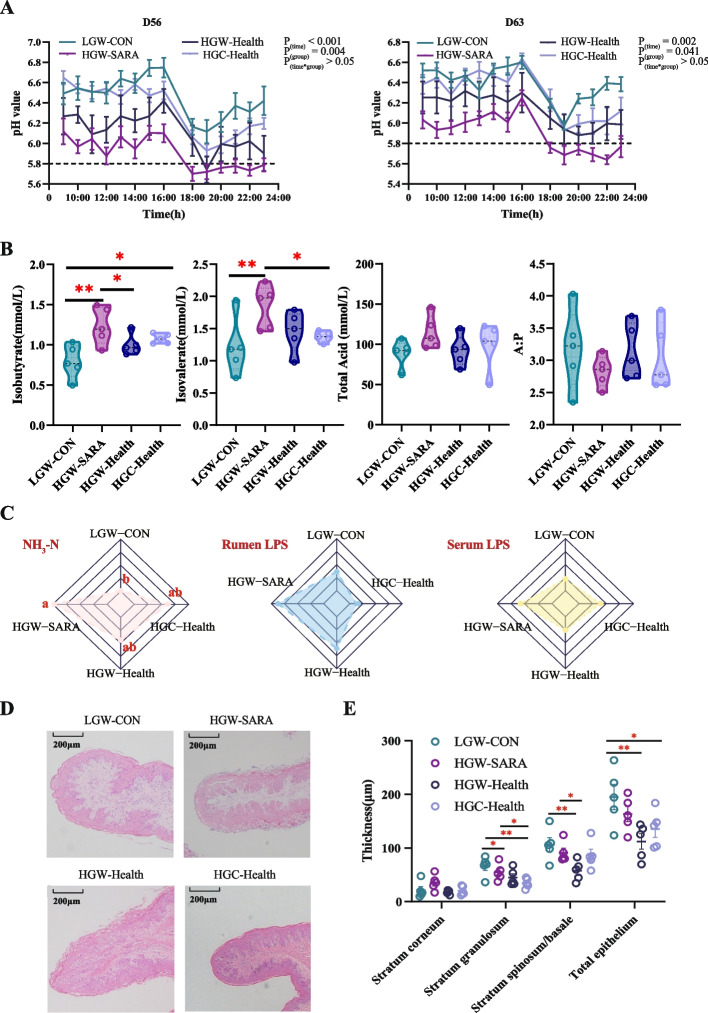
The comparison of the fermentation, the epithelium morphology, and thickness statistics in rumen among goats feeding low-grain whole corn control (LGW-CON), goats feeding high-grain whole corn and susceptible to SARA (HGW-SARA), goats feeding high-grain whole corn and tolerant to SARA (HGW-Health) and goats feeding high-grain crushed corn and tolerant to SARA (HGC-Health). **A** The dynamic change of pH values within 14 h after morning feeding were continuously monitored. The pH dynamic changes of rumen fluid were collected on day 56 of the trial period on the left and the pH dynamic changes on day 63 of the trial period on the right. **B** The concentrations of isobutyrate, isovalerate, and total volatile fatty acids and the ratio of acetate to propionate among LGW-CON, HGW-SARA, HGW-Health, and HGC-Health (*n* = 5 in each group). **C** The contents of NH_3_-N and lipolyaccharide in rumen, as well as contents of lipopolysaccharide in plasma among LGW-CON, HGW-SARA, HGW-Health, and HGC-Health goats. **D** The morphology of rumen epithelial papillae was observed by HE staining with magnification of × 100. From the inside to the outside were the lamina propria, the stratum basale, the stratum spinosum, the stratum granule, and the stratum corneum. **E** Rumen epithelium was a typical stratified squamous epithelium. The thickness of each layer of the epithelium was measured, and the differences in thickness among LGW-CON, HGW-SARA, HGW-Health, and HGC-Health goats were compared. Data of pH values (**A**), VFA concentrations (**B**), the contents of NH_3_-N and lipopolysaccharide (**C**), and the thickness of rumen epithelium (**E**) were expressed as the mean ± SEM and one-way ANOVA was performed, followed by LSD and DUNCAN test. *P < 0.05, **P < 0.01, ***P < 0.001 indicate significance

To investigate the impact of different proportions of rumen degradable starch on rumen fermentation, continuous pH monitoring was conducted in dairy goats for 14 h after morning feeding. The pH of high-grain whole corn SARA-susceptible group (HGW-SARA) goats remained below 5.8 for over 6 h on both days 56 and 63 of the trial period, surpassing the critical threshold for diagnosing SARA occurrence (*P* < 0.001, Fig. 1A; Additional file 1: Fig S1A-1B). In contrast, none of the healthy goats, including those in the low grain whole corn group (LGW-CON), high grain whole corn SARA-tolerant group (HGW-Health), and high grain crushed corn SARA-tolerant group (HGC-Health), met the criteria for SARA occurrence (Fig. 1A). Significantly higher concentrations of ruminal isobutyrate and isovalerate were detected in HGW-SARA goats than in LGW-CON goats and HGC-Health goats (*P* < 0.05, Fig. 1B). Compared with HGW-Health goats, only the concentration of isobutyrate was higher in HGW-SARA goats (*P* < 0.05, Fig. 1B). There were no significant differences in rumen VFAs ratio, acetate to propionate ratio, and other VFA concentrations among the four groups (*P* > 0.05, Fig. 1B; Additional file 1: Fig S2A-S2B). Additionally, when comparing the differences in NH_3_-N, and lipopolysaccharide (LPS) in the rumen and LPS in plasma, results showed that NH_3_-N concentration was significantly higher in HGW-SARA goats comparing to LGW-CON goats (*P* < 0.05), while there were no remarkable differences observed in the concentration of LPS within either the rumen or plasma samples (*P* > 0.05, Fig. 1C).

The HE-stained section of the rumen epithelial papilla and the statistics on epithelial thickness revealed significant shedding of stratum corneum cells in HGW-SARA goats (Fig. 1D). In comparison to LGW-CON goats, there were significant decreases in the thickness of stratum granulosum and total epithelium in HGW-Health and HGC-Health goats (*P* < 0.05) with tighter cell arrangement (Fig. 1D). Following the consumption of high-RDS diets, there was a reduction in the thicknesses of the stratum granulosum, stratum spinosum/basale, and total epithelium to varying degrees (*P* < 0.05, Fig. 1E).

### The rumen microbiome underwent a transformation in HGW-SARA and healthy dairy goats

**Fig. 2:**
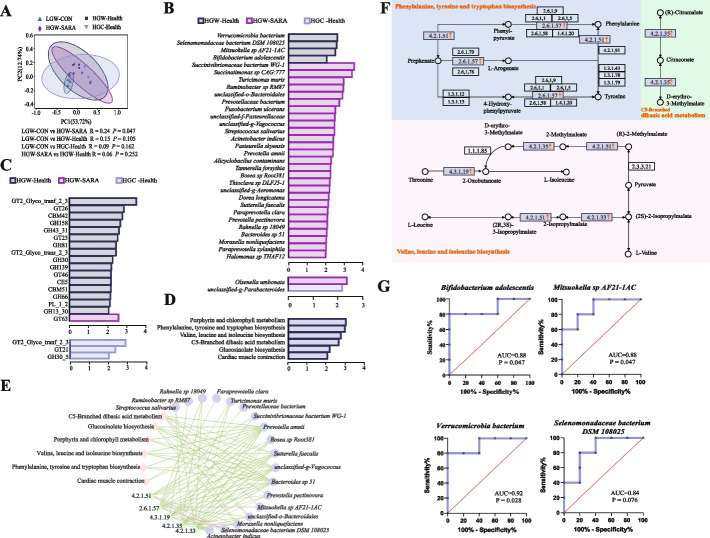
The differences of rumen microbial composition and functions between HGW-SARA and healthy goats (LGW-CON, HGW-Health, and HGC-Health). **A** The β diversity in PcoA of rumen microbe in LGW-CON, HGW-SARA, HGW-Health, and HGC-Health. **B** LEfSe analysis showing differential rumen bacteria in HGW-Health vs HGW-SARA goats and HGC-Health vs HGW-SARA goats (LDA > 2, *P* < 0.05). **C** The differential CAZy enzymes in HGW-Health vs HGW-SARA and HGC-Health vs HGW-SARA goats (LDA > 2, *P* < 0.05) according to LEfSe analysis. **D** The differential KEGG pathways at level 3 between HGW-Health and HGW-SARA goats (LDA > 2, *P* < 0.05) in Lefse analysis. **E** Association analysis in Spearman of differential bacteria with differential pathways and differential enzymes involved in these differential pathways, and only significant relationships (*P* < 0.05) were shown. **F** Showing 3 differential KEGG pathways, separately were phenylalanine, tyrosine and tryptophan biosynthesis, C5-Branched dibasic acid metabolism and valine, leucine, and isoleucine biosynthesis, and the KEGG enzymes participated in these pathways, including 4.2.1.51, 4.3.1.19, 2.6.1.57, 4.2.1.35, and 4.2.1.33. **G** The four increased species in the HGW-Health comparing to HGW-SARA goats were detected by the area under the curve (AUC) of the receiver operating characteristic (ROC) curve to explore their performance as a classification model for SARA-susceptibility and tolerance

The rumen microbiome metagenomic data revealed that the overall bacteria in rumen PcoA showed a significant separation between the cluster of HGW-SARA goats and LGW-CON goats (R = 0.24, *P* = 0.047). However, there was no significant difference in β diversity between HGW-SARA and high grain feeding healthy goats (Fig. 2A). LEfSe analysis revealed enrichment of 16 bacterial species in LGW-CON goats, notably belonging to genus *Prevotella* and phylum *Bacteroidetes* (LDA > 3, *P* < 0.05). On the other hand, 12 bacterial species were enriched in HGW-SARA goats with eight of which belonged to the phylum *Proteobacteria* (LDA > 3, *P* < 0.05), as shown in Additional file 1: Fig S3A. In comparison to LGW-CON goats, the relative abundance of GH24, GH25, and GH108 was significantly increased in HGW-SARA goats (*P* < 0.05, Additional file 1: Fig S3B). *Ruminococcus sp*, *Ruminococcus bromii*, and *Clostridiaceae bacterium* were highly correlated with GH24 and GH25 in HGW-SARA goats (Additional file 2: Table S1; Additional file 1: Fig S3C).

LEfSe analysis was conducted to compare differential bacteria of HGW-SARA and HGW-Health goats, and 31 differential bacteria was screened in LDA > 2, *P* < 0.05. *Verrucomicrobia bacterium*, *Selenomonadaceae bacterium DSM 108025*, *Mitsuokella sp AF21-1AC*, and *Bifidobacterium adolescentis* were enriched in HGW-Health goats. Comparing with HGW-SARA goats, *unclassified-g-Parabacteroides* was enriched in HGC-Health goats (Fig. 2B). It was found that only *Proteobacteria* and *Fusobacteria* of differential bacteria selected in species level were not enriched in the rumen of healthy dairy goats with high RDS (Additional file 1: Fig S4A). Comparing to HGW-SARA goats, GH43_31, GH30 and GH13_30 were significantly enriched in HGW-Health goats (LDA > 2, *P* < 0.05), while GH30_5 (LDA = 2.01, *P* = 0.028) was significantly enriched in HGC-Health goats (Fig. 2C). Phenylalanine, tyrosine and tryptophan biosynthesis, valine, leucine and isoleucine *biosynthesis*, C5-Branched dibasic acid metabolism, and others were significantly enriched in HGW-Health goats compared with HGW-SARA goats (LDA > 2, *P* < 0.05, Fig. 2D). However, there were no remarkable differences in the level 3 KEGG pathways between HGC-Health and HGW-SARA goats.

### The microbiome thriving in HGW-Health goats is linked to the metabolism and biosynthesis of amino acids

To further delve into the function and significance of rumen microbiome enriched in HGW-Health, we conducted a comparison of the variances in KEGG enzymes between HGW-Health and HGW-SARA goats. Additionally, a total of 56 KEGG enzymes were found to be enriched (LDA > 2, *P* < 0.05), with an impressive count of 50 being enriched in HGW-Health goats (Additional file 1: Fig S4B), contributing to essential roles across 29 KEGG pathways. Notably, specific enzymes such as 4.2.1.51 and 2.6.1.57 are involved in phenylalanine, tyrosine and tryptophan biosynthesis, and 4.3.1.19, 4.2.1.35, and 4.2.1.33 participated in valine, leucine, and isoleucine biosynthesis, respectively; and 4.2.1.35 is involved in C5-branched dibasic acid metabolism (Additional file 1: Fig S4C). Correlation analysis revealed significant associations between 18 species of bacteria and differential KEGG pathways, as well as related differential KEGG enzymes. Specifically, *Selenomonadaceae bacterium DSM 108025* showed positive correlations with 4.3.1.19, 4.2.1.51, and 2.6.1.57 (rho > 0.6, *P* < 0.05). Additionally, *Mitsuokella sp AF21-1AC* was positively associated with the enzyme activity of 2.6.1.57 (rho = 0.867,* P* = 0.003) and C5-Branched dibasic acid metabolism (rho = 0.644, *P* = 0.044, Fig. 2E). These findings were summarized in Fig. 2F, which shows that these enzymes primarily participated in amino acid biosynthesis such as tyrosine, L-leucine, L-isoleucine, and L-valine while also regulating Threonine metabolism.

Receiver operating characteristic (ROC) analysis was employed to ascertain whether the 4 species of bacteria enriched in HGW-Health goats could function as biomarkers for distinguishing between SARA-susceptible and SARA-tolerant goats on the same diets. It was revealed that 3 out of these 4 species were able to effectively diagnose and classify SARA-susceptible and SARA-tolerant goats with an AUC ≥ 0.88 (*P* < 0.05). Specifically, *Bifidobacterium adolescentis* and *Mitsuokella sp AF21-1AC* exhibited an AUC = 0.88, while *Verrucomicrobia bacterium* demonstrated an impressive AUC = 0.92 (Fig. 2G).

### Epithelial inflammation manifested in HGW-SARA dairy goats

**Fig. 3:**
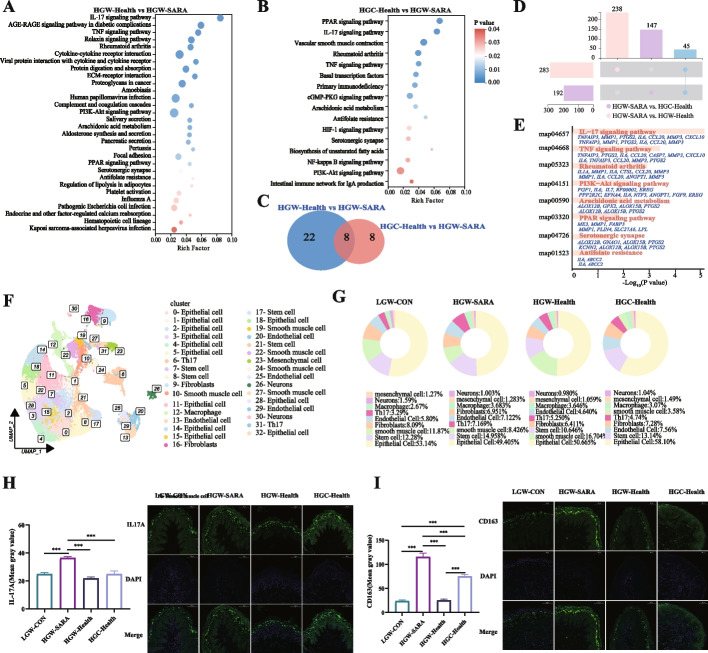
Effects of differences in susceptibility and tolerance to SARA on rumen epithelial inflammation. **A** Significantly different KEGG pathways of differential gene enrichment between HGW-SARA and HGW-health goats. **B** Significantly different KEGG pathways of differential gene enrichment between HGW-SARA and HGC-health goats. **C** Venn diagram showed the number of differential KEGG pathways and the number of unique KEGG pathways co-enriched by HGW-SARA vs HGW-Health and HGW-SARA vs HGC-Health. **D** The UpSet diagram showed that there were 283 significantly different genes between HGW-Health and HGW-SARA, and 192 significantly different genes between HGC-Health and HGW-SARA, of which 45 differentially expressed genes are present in these 2 comparing groups in common. Two hundred thirty eight existed only in the HGW-Health vs HGW-SARA, and 147 existed only in the HGC-Health vs HGW-SARA. **E** Eight KEGG pathways co-enriched in HGW-Health vs HGW-SARA and HGC-Health vs HGW-SARA and the differential genes involved in these pathways, the first line of which is the differential genes between the HGW-Health and HGW-SARA groups. The second line is the differential genes between HGC-Health and HGW-SARA groups. **F** The classification and clustering of 33 clusters obtained from the cluster results of single nuclear transcriptome sequencing after UMAP dimension reduction and the cell types of which were identified. **G** The composition of cells in rumen epithelium and the proportion of various types of cells in the four groups of LGW-CON, HGW-SARA, HGW-Health, and HGC-health. **H** Using IL-17A as marker gene, the distribution, and number of Th17 in rumen epithelial papillae of LGW-CON, HGW-SARA, HGW-Health, and HGC-Health were quantitatively detected by immunofluorescence. **I** Using CD163 as marker gene, the distribution and number of Macrophages in rumen epithelial papillae of LGW-CON, HGW-SARA, HGW-Health, and HGC-Health were quantitatively detected by immunofluorescence. Data of **H, I** expressed as the mean ± SEM and one-way ANOVA was performed, followed by LSD and DUNCAN test. *P < 0.05, **P < 0.01, ***P < 0.001 indicate significance

To further investigate whether alterations in the rumen microbiota influence the host healthy/unhealthy state, we conducted a comparative analysis of gene expression in the host rumen epithelium. Comparing to HGW-SARA goats, 124 genes exhibited downregulated expression and 159 genes showed upregulated expression in HGW-Health goats (*P* < 0.05, |log2FC|> 1, Additional file 1: Fig S5A). Meanwhile, when comparing HGC-Health goats to HGW-SARA goats, 123 genes were upregulated and 69 genes were downregulated (*P* < 0.05, |log2FC|> 1, Additional file 1: Fig S5B). Thirty divergent pathways between HGW-Health and HGW-SARA, as well as 16 differential pathways between HGC-Health and HGW-SARA were enriched (Fig. 3A and B). Notably, 8 pathways were shared across both comparisons (Fig. 3C) including the IL-17 signalling pathway, TNF signalling pathway, PI3K-Akt signalling pathway, and others (*P*_HGW-SARA vs HGW-Health_ < 0.01, *P*_HGW-SARA vs HGC-Health_ < 0.05, Additional file 2: Table S2; Fig. 3E). Delving further into the overlap between the two differential comparison sets, 45 genes were consistently differentially expressed (*P* < 0.05, |log2FC|> 1, Fig. 3D). Of particular interest, genes such as *TNFAIP3*, *MMP1*, *PTGS2*, *IL6*, and *CCL20*, which are implicated in the IL-17 signalling pathway, were concurrently downregulated in both HGW-Health and HGC-Health goats relative to HGW-SARA goats (Fig. 3E), highlighting a shared response potentially linked to inflammation and immune regulation.

Single-nucleus RNA sequencing was employed to elucidate the cellular constitution of the rumen epithelium. Thirty three distinct clusters were identified, with cluster 6 and cluster 31 being verified as T helper cell 17 (Th17) populations, and cluster 12 confirmed as Macrophages (Fig. 3F). A comparative assessment of the cellular composition in four groups uncovered an elevated proportion of Th17 cells specifically in HGW-SARA goats (7.17%), a marked increase over LGW-CON (3.29%), HGW-Health (5.25%), and HGW-Health (4.74%). Conversely, the macrophage population displayed a relatively consistent ratio across all four goat types (Fig. 3G). Given that *IL-17A* and *CD163* serve as established biomarkers for Th17 cells and macrophages, respectively, immunofluorescence assays were carried out to map their distribution and quantified their expression levels. The findings highlighted a substantial increase in *IL-17A* expression in HGW-SARA goats compared to LGW-CON, HGW-Health, and HGC-Health goats (*P* < 0.001, Fig. 3H). Despite the macrophage ratio remaining uniform across groups, *CD163* expression was notably augmented in HGW-SARA goats relative to LGW-CON, HGW-Health, and HGC-Health (*P* < 0.001), with HGC-Health also exhibiting significantly higher *CD163* expression compared to both HGW-Health and LGW-CON (*P* < 0.001, Fig. 3I).

### The proliferation and absorption capacity of rumen epithelial cells were enhanced in HGW-Health and HGC-Health

**Fig. 4:**
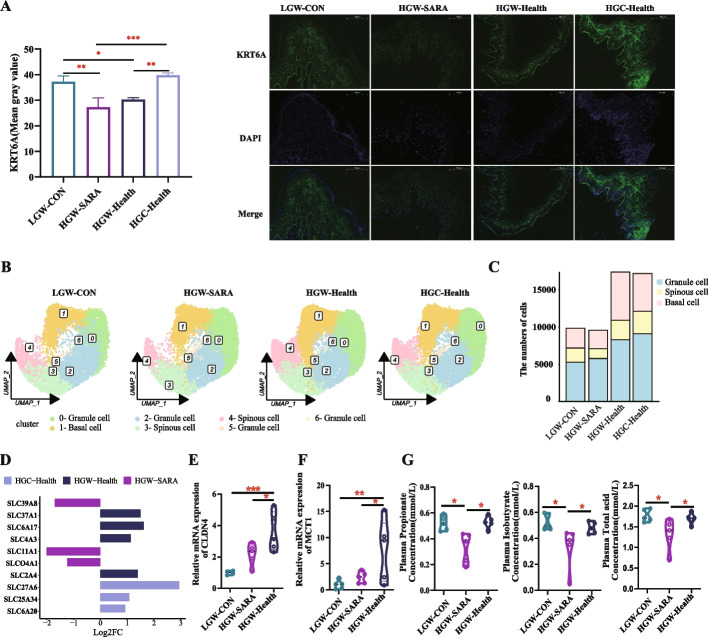
Effects of differences in susceptibility and tolerance to SARA on rumen epithelial barrier and absorption. **A** Using KRT16 as marker gene, the distribution, and number of epithelial cells in rumen epithelial papillae of LGW-CON, HGW-SARA, HGW-Health, and HGC-Health were quantitatively detected by immunofluorescence. **B** The epithelial cells were subdivided into subclusters and annotated, and the UMAP showed the clustering of each cluster in the four groups of LGW-CON, HGW-SARA, HGW-Health, and HGC-Health, and the cell types were noted. **C** The number of epithelial cells in LGW-CON, HGW-SARA, HGW-Health, and HGC-Health groups were analyzed, including granule cell, spinous cell, and basal cell. **D** The differential expression of solute carrier family genes in rumen epithelium showed only the genes with log2|FC|≥ 1 in HGW-SARA vs HGW-Health and HGW-SARA vs HGC-health. **E,F** The relative mRNA expression of *CLDN4* (**E**) and *MCT1* (**F**) of LGW-CON, HGW-SARA, and HGW-Health goats. **G** The concentrations of propionate, isobutyrate, and total acid of volatile fatty acids in plasma of LGW-CON, HGW-SARA and HGW-Health goats

An in-depth single-nucleus RNA analysis not only revealed the presence of Th17 and Macrophages but also delineated a broad cellular landscape, encompassing various cell types: Epithelial cells across twelve clusters (0, 1, 2, 3, 4, 5, 11, 14, 15, 18, 28, 32), Stem cells in four clusters (7, 8, 17, 21), Fibroblasts in two clusters (9, 16), Smooth muscle cells across five clusters (10, 19, 22, 24, 27), Endothelial cells in four clusters (13, 20, 25, 29), Mesenchymal cells in one cluster (23), and Neurons in two clusters (26, 30) (Fig. 3F). Remarkably, Epithelial cells constituted the majority, with their proportion notably augmented in LGW-CON (53.14%), HGW-Health (50.67%), and HGC-Health (58.10%) compared to HGW-SARA (49.41%) (Fig. 3G). Additionally, a stark contrast emerged in the total cellular count within rumen epithelium. Of note, the Epithelial cell counts nearly doubled in HGW-Health and HGC-Health goats relative to LGW-CON and HGW-SARA (Additional file 2: Table S3). To validate these findings, immunofluorescence staining employing *KRT6A*, a prominent epithelial marker, disclosed a significant upregulation in HGC-Health goats vis-a-vis HGW-SARA (*P* < 0.001, Fig. 4A). Delving into the underlying cause of this epithelial cell discrepancy, a subcluster classification of epithelial cells was conducted. The UMAP analysis segregated seven subclusters, with their top three marker genes illustrated in Additional file 1: Fig S6, categorizing epithelial cells into Granule cells (clusters 0, 2, 5, 6), Spinous cells (3, 4), and Basal cells (1) (Fig. 4B). A conspicuous variation in Spinous and Basal cell counts across the groups was discernible. Quantification and proportion analysis confirmed Granule cells as the predominant type, but Basal and Spinous cells exhibited the most substantial numeric disparity, corroborating the UMAP visualization (Fig. 4C; Additional file 2: Table S4).

The expression levels of genes in solute carrier family between HGW-SARA and HGW-Health goats were analyzed, as well as between HGW-SARA and HGC-Health goats. The result showed that the expression levels of certain solute carrier gene families were markedly elevated in the HGW-Health and HGC-Health dairy goats (log_2_FC** ≥ **1, *P* < 0.05, Fig. 4D). Reflecting on epithelial barrier function, *CLDN4* mRNA expression was significantly enhanced in HGW-Health goats compared to LGW-CON and HGW-SARA (Fig. 4E). Similarly, *MCT1*, indicative of epithelial absorption capacity, was significantly upregulated in HGW-Health goats (Fig. 4F). Lastly, plasma VFAs assessment revealed that propionate, isobutyrate, and total VFAs concentrations were significantly reduced in HGW-SARA goats compared to LGW-CON and HGW-Health, with no significant variance noted in other VFAs or their ratios among all groups (Fig. 4G; Additional file 1: Fig S7A-B). HGC-Health goats did not exhibit significant deviations in either VFAs concentrations or ratios from LGW-CON and HGW-SARA goats (Additional file 1: Fig S7C-D), underscoring the complex interplay of factors influencing epithelial dynamics and metabolic profiles.

### Differences in ruminal metabolite profiles between HGW-SARA and healthy dairy goats

A nontargeted metabolomics approach was adopted to contrast HGW-SARA against its healthy counterparts. A distinct separation between LGW-CON goats and those fed high-RDS diets, albeit without clear differences among HGW-SARA, HGW-Health, and HGC-Health goats (Additional file 1: Fig S8A). A permutation test with a Q^2^Y intercept less than 0.05 and an R^2^Y intercept less than 0.3 confirmed the absence of model overfitting, attesting to the model’s robustness and reliability (Additional file 1: Fig S8B).

Compared to those in LGW-CON goats, 37 metabolites were significantly altered (VIP > 1, FDR < 0.05), with 17 elevated and 20 diminished in HGW-SARA goats (Additional file 1: Fig S8C). These alterations were implicated in ten KEGG pathways, separately being alpha-Linolenic acid metabolism, cAMP signalling pathway, renin secretion, regulation of lipolysis in adipocytes, and others (Additional file 2: Table S5; Additional file 1: Fig S8D).

**Fig. 5:**
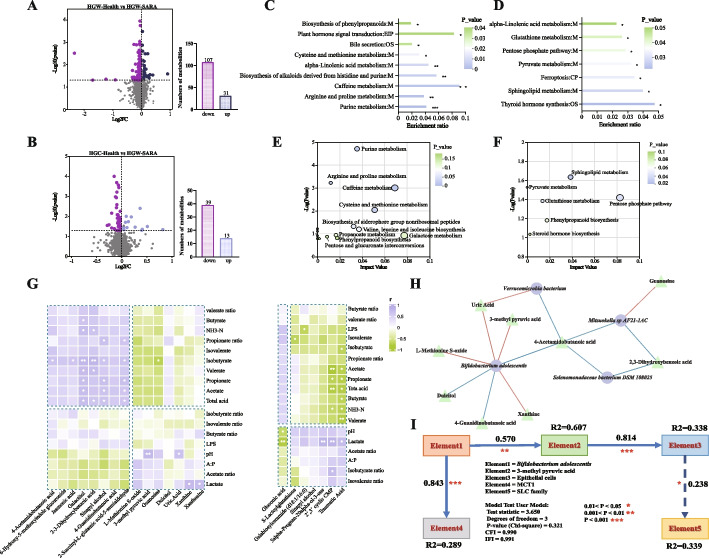
The differences of metabolites in the rumen of HGW-SARA, HGW-Health, and HGC-health goats. **A** Volcanic maps showed that 107 metabolites were significantly down-regulated and 31 metabolites were significantly upregulated in the rumen of HGW-Health goats compared with HGW-SARA goats. **B** Volcanic maps showed that 39 metabolites were significantly downregulated and 13 metabolites were significantly upregulated in the rumen of HGC-Health goats compared with HGW-SARA goats. **C,D** The KEGG pathways significantly enriched by differential metabolites between HGW-Health and HGW-SARA (**C**), between HGC-Health and HGW-SARA (**D**). **E,F** The bubble diagram showed the metabolic pathways enriched by KEGG topological analysis between HGW-Health and HGW-SARA (**E**), between HGC-Health and HGW-SARA (**F**). **G** The differential metabolites involved in the pathway obtained by KEGG enrichment analysis and topological analysis were screened, and their correlation with rumen fermentation parameters and concentrations of lactate, lipopolysaccharide, and NH_3_-N was analyzed by Pearson correlation index. **H** Differential bacteria significantly enriched to HGW-Health comparing to HGW-SARA were screened, and their association with differential metabolites was analyzed by Pearson correlation index. The network diagram only showed the part of significant association. **I** Structural equation models were used to explore causal relationships between key microbiome, key metabolites, solute carrier families, epithelial resorption related genes, and epithelial cell numbers utilizing the lavaan package in the R software environment

Further comparisons revealed 138 differentially abundant metabolites between HGW-SARA and HGW-Health goats, 107 of which were upregulated in HGW-SARA goats and 31 in HGW-Health goats (VIP > 1, *P* < 0.05, Fig. 5A). Between the HGW-SARA and HGC-Health groups, 52 differentially abundant metabolites emerged, 39 of which were elevated in the HGW-SARA group and 13 in the HGC-Health group (VIP > 1, *P* < 0.05, Fig. 5B). Cysteine and methionine metabolism, alpha-Linolenic acid metabolism, Arginine and proline metabolism, Purine metabolism, and Bile secretion were enriched in the comparison between HGW-SARA and HGW-Health (*P* < 0.05, Fig. 5C). For HGW-SARA versus HGC-Health, alpha-Linolenic acid metabolism, Glutathione metabolism, Pyruvate metabolism, and Thyroid hormone synthesis were significantly highlighted (*P* < 0.05, Fig. 5D). To delve deeper, KEGG topological analysis illuminated metabolic discrepancies. Between HGW-SARA and HGW-Health, Cysteine and methionine metabolism, valine, leucine, and isoleucine biosynthesis, Arginine and Proline metabolism, and other 12 pathways were distinguished (Fig. 5E). Meanwhile, Pentose phosphate pathway, Pyruvate metabolism, and other 4 pathways holding notable impact in the comparison between HGW-SARA and HGC-Health goats (Fig. 5F).

### Regulatory functions of the ruminal microbiome and metabolites in rumen epithelial absorption

Correlation analysis was employed to elucidate relationships between these metabolites and key rumen parameters such as fermentation products, the concentration of LPS, NH_3_-N, and lactate. The results showed that metabolites elevated in HGW-SARA goats compared to HGW-Health were positively correlated with increased VFAs and NH_3_-N concentrations. Notably, 3-methyl pyruvic acid (rho = 0.767, *P* = 0.001) were significantly positively correlated with the ruminal pH. When HGW-SARA was compared with HGC-Health, the upregulated metabolites in HGW-SARA demonstrated stronger positive associations with lactate and pH and inverse relationships with VFAs levels (Fig. 5G).

To investigate how microbiome and metabolites influence the SARA tolerance of HGW-Health goats, correlations were examined between HGW-Health-enriched microbiome and metabolites integral to metabolic regulation. *Bifidobacterium adolescentis* emerged as a pivotal microbe, displaying strong positive correlations with metabolites like 3-methyl pyruvic acid (rho = 0.733, *P* = 0.021), L-Methionine S-oxide (rho = 0.758, *P* = 0.016), uric acid (rho = 0.745, *P* = 0.018), and Xanthine (rho > 0.6, *P* < 0.05, Additional file 2: Table S6; Fig. 5H).

Concomitantly, both metagenomic and metabolomic data pointed to enriched valine, leucine, and isoleucine biosynthesis pathways in the HGW-SARA versus HGW-Health comparison. With a focus on the pivotal metabolite 3-methyl pyruvic acid involved in this pathway and the differential bacterium *B. adolescentis*, a structural equation model (SEM) was constructed to explore the potential interplay among five key elements: *B. adolescentis*, 3-methyl pyruvic acid, SLC family genes, *MCT1*, and epithelial cell proliferation. The model suggested a putative pathway in which *B. adolescentis* may influence *MCT1* expression and mediate the effect of 3-methyl pyruvic acid on epithelial cell proliferation, potentially contributing to the maintenance of epithelial integrity (Fig. 5I).

### Cross-transplantation of the rumen microbiome alleviated SARA and epithelial inflammation

**Fig. 6:**
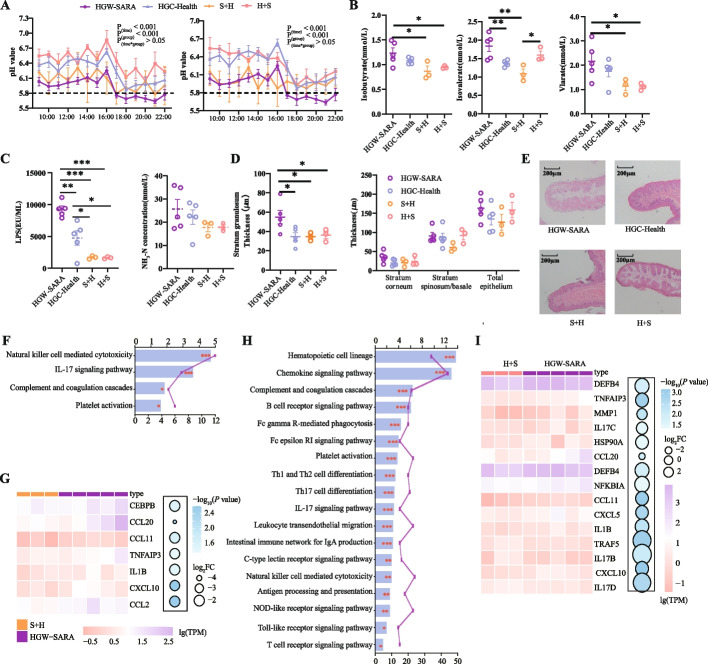
After cross-transplantation of rumen contents, dairy goats showed different rumen fermentation patterns and different occurrence of epithelial inflammation. **A** The dynamic change of pH values within 14 h after morning feeding were continuously monitored. The pH dynamic changes of rumen fluid were collected on day 7 after transplantation on the left and the pH dynamic changes on day 14 after transplantation on the right. **B** The concentrations of isobutyrate, isovalerate, and valerate significantly changed among the HGW-SARA (*n* = 5), HGC-Health (*n* = 5), H + S (*n* = 3), and S + H (*n* = 3) groups. **C** Contents of NH_3_-N and lipopolysaccharide in the rumen of HGW-SARA, HGC-Health, H + S, and S + H goats. **D** The thickness of each layer of the rumen epithelium, including the stratum granule, stratum corneum, stratum spinosum/basale and total epithelium, among HGW-SARA, HGC-Health, H + S, and S + H goats. **E** HE staining revealed morphological differences in the rumen epithelial papillae at a magnification of 100 × among HGW-SARA, HGC-Healthy, H + S, and S + H goats. **F** Transcriptomic analysis revealed differential KEGG pathways associated with the immune system in the rumen epithelial between the HGW-SARA and S + H groups. **G** Heatmap showing the expression of DEGs involved in the IL-17 signalling pathway in each sample from HGW-SARA and S + H goats, with a bubble map showing the log_2_FC values. **H** Transcriptomic analysis revealed differential KEGG pathways associated with the immune system in the rumen epithelial between the HGW-SARA and H + S groups. **I** Heatmap showing the expression of DEGs involved in the IL-17 signalling pathway in each sample from HGW-SARA and H + S goats, with a bubble map showing the log_2_FC values

To negate host-specific influences, an interventional study involving rumen content transplantation was executed to elucidate the roles of microbiome and metabolites in rumen function. Rumen fluids from three HGW-SARA goats were transplanted into HGC-Health goats, labelled S + H, while fluids from three HGC-Health goats were transferred to HGW-SARA goats, designated H + S (Additional file 1: Fig S1B). Monitoring rumen pH at the 7th and 14th day post-transplantation revealed that only the HGW-SARA goats maintained a pH below 5.8 for more than 3 h; in contrast, both the S + H and H + S groups exhibited higher pH values (Fig. 6A). In VFA profiles, S + H and H + S goats showed significantly reduced isobutyrate and valerate concentrations compared to HGW-SARA goats (*P* < 0.05). Isovalerate levels were significantly decreased in HGC-Health and S + H goats relative to HGW-SARA (*P* < 0.01), whereas other VFAs remained comparable across all groups (Fig. 6B). Notably, LPS content was significantly higher in HGW-SARA goats compared to HGC-Health, S + H, and H + S (*P* < 0.01), with S + H and H + S goats showing reduced LPS levels compared to HGC-Health (*P* < 0.05), while NH_3_-N levels showed no significant variation among the groups (Fig. 6C). Post-mortem assessments of rumen epithelial papillae indicated a significantly thicker stratum granulosum in HGW-SARA goats compared to HGC-Health, S + H, and H + S goats (*P* < 0.05, Fig. 6D). Clear detachment of the stratum corneum and widened gaps between layers were visually confirmed in HGW-SARA goats (Fig. 6E).

Transcriptomic profiling of rumen epithelium in HGW-SARA vs. S + H and HGW-SARA vs. H + S comparisons aimed to elucidate the mechanisms underlying the influence of the microbiome and metabolites on SARA-susceptibility and tolerance. KEGG analysis revealed four enriched immune-related pathways in HGW-SARA vs. S + H, including Natural killer mediated cytotoxicity and IL-17 signalling pathway (*P* < 0.001, Fig. 6F). Seven genes enriched in the IL-17 pathway, such as *CCL20*, *IL1B*, *CXCL10*, and *CCL2* were upregulated in HGW-SARA (*P* < 0.05, |log2FC|> 1, Fig. 6G). At the same time, HGW-SARA vs. H + S comparison revealed 18 immune-related pathways enriched, among which Th17 Cell Differentiation, and IL-17 signalling pathway stood out (*P* < 0.001, Fig. 6H). Fifteen differentially expressed genes were identified in IL-17 signalling pathway, with genes *TRAF5* and *IL17B* upregulated in H + S goats, while other pro-inflammatory genes were upregulated in HGW-SARA goats (*P* < 0.05, |log2FC|> 1, Fig. 6I).

### Cross-transplantation of the rumen microbiome altered the composition and function of the rumen microbiota and its metabolic pattern

The PCoA of rumen microbiome sequences among HGW-SARA, HGC-Health, S + H, and H + S goats revealed a significant difference between HGW-SARA and S + H goats (R = 0.354, *P* = 0.047), although no marked differences were detected in the other group comparisons (*P* > 0.05, Additional file 1: Fig S9A). LEfSe revealed that HGW-SARA goats harbored enriched populations of *Succinivibrionaceae bacterium WG-1*, *Succinatimonas sp CAG 777*, *Ruminoccocus bromii*, and *Ruminoccocus sp* (LDA_HGW-SARA vs H+S_ > 3, LDA_HGW-SARA vs S+H_ > 3, *P* < 0.05) compared to both H + S and S + H goats. Conversely, H + S and S + H goats displayed elevated levels of *Bacteroidales bacterium*, *Prevotella sp ne3005*, and *unclassified-g-Prevotella* (LDA_HGW-SARA vs H+S_ > 3, LDA_HGW-SARA vs S+H_ > 3, *P* < 0.05) relative to HGW-SARA goats (Additional file 1: Fig S9B). Functional profiling of the microbiome revealed that pathways such as Cysteine and methionine metabolism and Pyrimidine metabolism (LDA > 3, *P* < 0.05) to be more abundant in HGW-*SARA* compared to S + H goats. S + H goats, on the other hand, showed enrichment in pathways like Quorum sensing, Two-component system, Hippo signalling pathway—multiple species (LDA > 3, *P* < 0.05), with Two-component System also enriched in H + S goats compared to HGW-SARA (LDA = 3.21, *P* < 0.05, Additional file 1: Fig S9C). Comparative analysis of CAZymes via LEfSe indicated differential enrichment. For instance, CE10, CE15, and CE12 (LDA > 4, *P* < 0.05) were more prevalent in S + H goats, while GT92 (LDA = 5.00, *P* < 0.05) was dominant in HGW-SARA when comparing HGW-SARA to S + H. A similar pattern emerged with additional CAZymes when contrasting HGW-SARA with H + S goats, which found that GT92, GT2_Glyco_traf_2_4 (LDA > 4, *P* < 0.05) were enriched in HGW-SARA and GH64 (LDA = 4.51, *P* < 0.05) was enriched in H + S (Additional file 1: Fig S9D). Nine bacterial species enriched in H + S and S + H goats relative to HGW-SARA were classified across various genera and phyla, including *Mycoplasma*, *Prevotella*,* unclassified-c-Clostridia*, *unclassified-c-Opitutae*, *unclassified-c-Bacteroidales*, *unclassified-c-Clostridiales*, and *unclassified-c-Verrucomicrobia*, as well as the phyla Bacteroidetes, Firmicutes, Tenericutes, and Verrucomicrobia (Additional file 1: Fig S9E).

PLS-DA effectively discriminated between HGW-SARA and both S + H and H + S groups (Additional file 1: Fig S9F). KEGG enrichment analyses highlighted 12 pathways in HGW-SARA vs S + H, with 7 pertaining to metabolism, including galactose metabolism, arginine and proline metabolism, and tryptophan metabolism (*P* < 0.05). Similarly, 14 pathways were enriched in HGW-SARA vs H + S, 5 of which were metabolic, such as tryptophan metabolism, glycosaminoglycan biosynthesis—chondroitin sulfate/dermatan sulfate, and glycerophospholipid metabolism (*P* < 0.05, Additional file 1: Fig S9G). Notably, tryptophan metabolism was a common feature enriched in comparisons of HGW-SARA with both S + H and H + S goats. Analysis of specific metabolites in this pathway showed decreased 2-Formaminobenzoylacetate and increased N-Acetylserotonin and Xanthurenic Acid in H + S and S + H goats compared to HGW-SARA (Additional file 2: Table S7).

### *Bifidobacterium adolescentis* participated in the tolerance of dairy goats to SARA

**Fig. 7:**
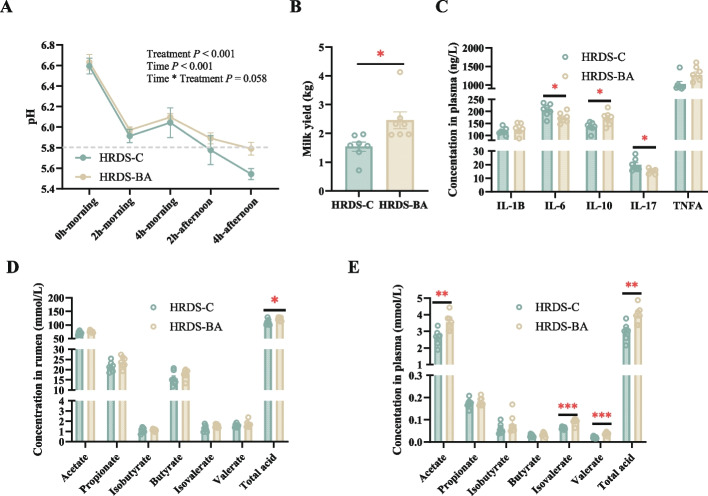
Through feeding *Bifidobacterium adolescentis*, HRDS-C and HRDS-BA dairy goats exhibited different rumen fermentation and *Bifidobacterium adolescentis* helped VFAs absorption and inflammation relieve. **A** The dynamic change of pH values in the 2 and 4 h after morning and afternoon feeding were continuously monitored. **B** The differences of milk yield between HRDS-C and HRDS-BA goats. **C** concentrations of IL-1β, IL-17, IL-6, IL-10, and TNF-α in the plasma. **D,E** The concentration of VFAs in rumen (**D**) and in plasma (**E**), including acetate, propionate, isobutyrate, butyrate, and total acid

To explore the function of rumen microbiome to prevent SARA, we also constructed a SARA model of 14 dairy goats by feeding the same high RDS diets, and randomly selected 7 dairy goats to supplementarily feed *B. adolescentis*, which was identified as the key microbiome to help the host maintain SARA tolerance. After continuously monitoring pH in the 42nd day of the trial period, the pH of HRDS-BA dairy goats did not meet the criteria of SARA while the pH of HRDS-C dairy goats was lower than 5.8 over 3 h (*P* < 0.05, Fig. 7A). The milk yield of HRDS-BA goats was significantly higher than HRDS-C goats (*P* < 0.05, Fig. 7B). IL-6 and IL-17 were significantly decreased in HRDS-BA goats, while IL-10 increased significantly in HRDS-BA goats (*P* < 0.05, Fig. 7C). The concentration of total VFAs in rumen was higher in HRDS-BA goats (*P* < 0.05) with no other differences of ruminal VFA concentration between HRDS-C and HRDS-BA goats (Fig. 7D). However, compared with HRDS-C, the concentrations of acetate, isovalerate, valerate, and total acids in HRDS-BA goats were significantly increased (*P* < 0.01, Fig. 7E).

## Discussion

The ruminal microbiome plays important roles in carbohydrate digestion and SCFA absorption, which provided 60–70% of the metabolizable energy for promoting milk yield and milk quality. The high-concentrate diet can lead to the more efficient energy conversion pattern: propionate-type fermentation, as well as the increased risks of the SARA [37, 38]. Our study is one of the few studies that systemically identified altered ruminal microbiome, metabolome, host ruminal transcriptome, and ruminal single nucleus transcriptome in response to high RDS feeding and its associated mechanisms in both regulating the SARA occurrence and SARA tolerance.

First, our study confirmed that dairy goats with a high-RDS diets will lead to the decrease of ruminal pH, increase of ruminal LPS, and activation of ruminal inflammation, as reported in other ruminants [13, 15, 27]. In the previous studies, it has been reported that the variation in the expression of the TLR/MyD88-NFκB pathway genes in the rumen epithelial wall significantly changed in steers with subacute ruminal acidosis [28, 39, 40]. In line with these previous studies, our rumen epithelial transcriptome data revealed that, compared to both tolerant groups, HGW-SARA was enriched in pathways including the IL-17 signalling pathway and TNF signalling pathway, Moreover, expression of inflammation-related genes such as *TNFAIP3*, *MMP1*, *PTGS2*, *IL6*, *CCL20*, and *MMP3* was upregulated in HGW-SARA, indicating the occurrence of epithelial inflammation as well. Furthermore, the nonspecific innate immune processes mediated by LPS stimulation and activated TLR/MyD88-NFκB pathway may not fully account for the mechanisms underlying the inflammatory response in dairy goats experiencing SARA [41]. Our single-nucleus transcriptomic analysis suggested an increase in Th17 cells and macrophages within HGW-SARA; notably, the marker genes, IL-17A for Th17 and CD163 for macrophages, were significantly elevated in HGW-SARA. In the previous studies, the activation of IL-17 signalling pathway and Th17 cell differentiation were shown to mediate the regulation of intestinal inflammation in mammals [29–31]. Mechanistically, inflammatory Th17 cells concomitantly express cytokines such as IL-17, IL-22, TNF-α, and IFN-γ, which activate the TLR/MyD88-NFκB pathway, thereby instigating an inflammatory response in the gastrointestinal epithelium [42–45]. Hence, our findings lead to the speculation that inflammation in SARA goats can be initiated through promotion of Th17 cell differentiation, activation of the IL-17 signalling pathway, and subsequent release of inflammatory cytokines to induce ruminal inflammation.

Second, some previous studies explored the linkage between the increased ruminal starch-degrading bacteria and their increased VFAs production in SARA [46, 47]. In line with these studies, our study revealed that the SARA dairy goats exhibit microbiome changes identified by a significant decrease in the phylum of Bacteroidetes and specifically decreases in the genus *Prevotella*, as well as a significant increases in the phylum Firmicutes and Proteobacteria and specifically the genera *Ruminococcus*. Further, according to the previously published work, these significantly enriched phyla and genera are prominent starch-degrading bacteria that promoted propionic acid-type fermentation [48–52]. Notably, in one previous study, some species of *Prevotella* have been previously identified as starch-degrading bacteria and significantly increasing in the dairy cows with SARA [53]. These differences may be contributed to significant differences of microbiome composition between cows and goats, and also the differential sequencing methods [54, 55]. However, several published researches also identified that the significant increase in family of *Ruminococcaceae* and genus *Ruminococcus* was accompanied with the SARA occurrence in cows and goats [56, 57]. Likewise, the genus *Prevotella* has also been previously identified as the main cellulolytic bacteria in mammals feeding on a plant-based diet [58, 59]. Hence, the significantly decreased *Prevotella* and the other cellulolytic bacteria, such as *Fibrobacter succinogenes* and *Butyrivibrio fibrisolvens*, in SARA goats in our present study and previous study [60, 61] may suggest significantly decreased fiber digestion ability when SARA occurs [62]. By further deeply analyzing the enzyme changes based on the CAZy database, there is an increase in the expression of genes (GH24 and GH25) encoding lipopolysaccharide synthases and lysases. Hence, from a metagenome insight, our study suggested that the increased of lipopolysaccharide synthases and lysases genes could serve as the underlined mechanism that induced the lysis of cellulolytic bacteria and increase of ruminal lipopolysaccharide concentration under low ruminal pH condition.

Thirdly, our study confirmed that dairy goats have significant individual differences in susceptibility and tolerance to a high-RDS diets as reported in cows and meat sheep, while providing a mechanistic understanding [20]. It is noticeable that goats developed SARA even under low level (21.17%) of RDS, while some goats were tolerant under high level (26.72%) RDS. In the previous studies, the proportion of fiber-degrading bacteria was greater in the rumen of SARA-tolerant cows when compared with the SARA-susceptible cows [15, 27]. Similar with these studies, our study proved that the SARA-tolerant goats were enriched with ruminal microbial enzymes GH43_31 and GH30, both associated with hemicellulose degradation [63, 64], indicating an intact cellulose digestion capability in SARA-tolerant ruminants. Further, it has also been reported that the variation in the expression of the Toll-like receptor genes *TLR2* and *TLR4* in the rumen epithelial wall significantly changed in steers with differential susceptibility to subacute ruminal acidosis [39, 40]. As opposed to goats with SARA, the SARA-tolerant goats can maintain the ruminal environment stable (with stable pH and less lipopolysaccharide when compared with the LGW-CON group) and avoid the Th17 cell-mediated ruminal inflammation [41], which were both identified in the present and previous studies [14, 18, 19, 32]. Notably, the β-diversity analysis has suggested the ruminal microbiome of SARA-tolerant goats is more similar to that of control goats fed with low-concentrate diets, rather than resembling the rumen microbiome of goats experiencing SARA. These results suggest that the ruminal microbiome is involved in the regulation of SARA tolerance. Herein, the exchanged introduction of rumen contents between SARA-susceptible goats and SARA-tolerant goats using RMT confirmed that the ruminal microbiome of SARA-tolerant goats triggered stable ruminal pH and a decrease of ruminal inflammation. Our further microbiome KEGG analysis indicated that SARA-tolerant goats exhibited significantly enhanced microbial amino acid biosynthesis ability, with significantly increased enzymes in the EC categories 4.2.1.51, 2.6.1.57, 4.3.1.19, 4.2.1.35, and 4.2.1.33 and enriched pathways of C5-Branched dibasic acid metabolism, valine, leucine, and isoleucine biosynthesis, phenylalanine, tyrosine, and tryptophan biosynthesis. Hence, these findings indicate that the rumen microbiome contribute to avoid the occurrence of ruminal inflammation and can help maintain the ruminal environment, partly through enhanced the amino acid biosynthesis.

Notably, according to the RMT results, our study identified that SARA-tolerant goats can also maintain stable ruminal pH and avoid the ruminal inflammation when received RMT from SARA-susceptible goats and fed with high RDS diets, but the underlying mechanism remains unknown. Our single-nucleus transcriptomic analysis uncovered an almost two-fold increase in epithelial cells within the SARA-tolerant groups when compared with SARA-susceptible and healthy control goats. Upon further examination of epithelial cell subsets, basal cells and spinous cells displayed the most prominent increases, with these two cell types being primarily responsible for the absorption of volatile fatty acids (VFAs) [41, 65]. Analyzing the expression of genes related to absorption and barrier function in the rumen epithelium, as well as solute carrier family genes, revealed significant upregulation of *MCT1*, *CLDN4*, *SLC37A1*, *SLC6A17*, *SLC4A3*, *SLC2A4*, *SLC27A6*, *SLC25A34*, and *SLC6A20* in SARA-susceptible goats. The basal cells and spinous cells have been suggested to serve as the main epithelial cells that response to the VFAs absorption [66], which can help reduce the ruminal VFAs accumulation and maintain ruminal pH stable [20]. Further, one recent study has suggested that the cattle adapted to the starch-rich diet exhibited greater rates of carbohydrate degradation, leading to increased total VFAs concentration. Meanwhile, these cattle adapted to the starch-rich diet also exhibited a greater increase in the rate of VFAs absorption than production [65]. To sum up, our present study and the previous studies all indicated that SARA-tolerant individuals exhibit increased production of VFAs by rumen microbiome, and enhanced proliferation of basal cells and spinous cells and their VFAs absorption.

Lastly, in the present study, compared to goats experiencing SARA, ruminal microbiota of HGW-Health goats showed a significant increase in the abundance of *B. adolescentis* which can serve as biomarkers for identifying SARA-tolerant individuals via ROC curve. Further, the microbiome and metabolome analyses enhanced nutrient synthesis in SARA-tolerant goats’ rumen, via valine, leucine, and isoleucine biosynthesis. Key metabolite, 3-methyl pyruvic acid as an intermediate product for the synthesis of isoleucine and valine, was upregulated in HGW-Health goats, correlating positively with pH, indicating its role in SARA tolerance regulation. Metagenomic analysis showed that the enzyme 4.3.1.19 for synthesizing 3-methyl pyruvic acid was also upregulated in the HGW-Health goats, fully demonstrating that microbial metabolism is involved in this pathway to promote the synthesis of branched-chain amino acids. Furthermore, *B. adolescentis* was also found to have a significant positive correlation with 3-methyl pyruvic acid. SEM showed *B. adolescentis* impacts epithelial cell proliferation, possibly via 3-methyl pyruvic acid regulation and influences *MCT1* expression, enhancing absorption. In the previous studies, *B. adolescentis* has been suggested as having the capacity to reduce the gut inflammation by inhibiting NF-κB activation and lipopolysaccharide production by gut microbiome [67]. Meanwhile, the *B. adolescentis* can also enhance the intestinal barrier through promoting the epithelial cell proliferation [68]. These results indicate that *B. adolescentis* has the potential to contribute to the SARA tolerance. Based on these findings, we further tested the roles of *B. adolescentis* in preventing SARA under high RDS diets. Subsequent results show that dietary supplementation of goats with *B. adolescentis* can help maintain ruminal pH and reduce ruminal inflammation through promoting VFAs absorption. Overall, the most important finding in the present study is the distinct ruminal microbiome found in SARA-tolerant goats, especially the significant increase in abundance of *B. adolescentis*, which were confirmed as being involved in the regulation of and promotion of rumen epithelial VFAs absorption.

## Conclusions

The results of this study revealed that high RDS diets lead to rumen epithelial inflammation in SARA susceptible goats, accompanied by the proliferation of Th17 and the activation of IL-17 signalling pathway, with a lower abundance of rumen gram-negative bacteria and fiber-degrading bacteria and a more unsatisfactory fiber degradation ability. However, the abundance of *B. adolescentis* in the rumen of SARA-tolerant dairy goats under high RDS diets can help maintain rumen homeostasis by using fermentation substrates to synthesize branched-chain amino acids and promoting epithelial cell proliferation and epithelial VFAs absorption. Combined with multi-omics analysis techniques, including single-nucleus transcriptome analysis, we clarify the mechanism of SARA occurrence and SARA tolerance in dairy goats, and find the key bacteria *B. adolescentis* regulates SARA tolerance and enhances lactation performances as verified through gavage to dairy goats, providing a solid theoretical basis for preventing and treating SARA. However, further research is required to verify the underlying mechanisms. Consequently, our comprehensive study has highlighted the immense potential of modulating VFAs absorption in the rumen epithelium to prevent metabolic diseases and improve production performance as a pivotal research area and a burgeoning hotspot in the field. This approach not only promises to enhance the efficiency of rumen fermentation, but also holds the key to maintaining the stability and integrity of the rumen environment, thereby contributing to sustainable livestock production to meet increasing consumer demands.

## Methods

### Ethics approval statement

This experiment was conducted at the Animal Research and Technology Center of Northwest A&F University (Yangling, Shaanxi, China), and it was performed in accordance with the recommended guidelines from the Administration of Affairs Concerning Experimental Animals (Ministry of Science and Technology, China, revised 2004). The protocol was approved by the Institutional Animal Care and Use Committee at Northwest A&F University.

### Animals and experimental design

#### SARA-susceptibility and tolerance of dairy goats’ model construction

Forty-seven ruminally cannulated, healthy, multiparous, but not presently pregnant and not presently lactating dairy goats (with body weights of 40 ± 4 kg) were recruited for the experiment. Feeding and management were described as follows: first, the animals were randomly divided into 2 groups and housed individually in their tie stalls: the LGW group (*n* = 11) was fed a low-grain diet (30% concentrate in which whole corn was the main energy feed) called LGW-CON, and the HGW group (*n* = 36) was fed a high-grain diet (70% concentrate in which whole corn was the main energy feed) for 35 days (Additional file 2: Table S8). Then, on the 28th and 35th days, the pH values of the ruminal fluids were measured every hour for 14 consecutive hours after feeding in the morning to monitor SARA occurrence (the pH was lower than 5.8 for more than 3 h) in dairy goats. The pH of the ruminal fluids was measured using a mobile pH meter (Ohaus Instruments Co. Ltd., China). On the 35th day, 15 dairy goats were identified as SARA susceptible goats and 21 dairy goats were identified as SARA-tolerant goats. We further increased the content of rumen-degradable starch (RDS) in the feed by modifying the corn processing method, aiming to investigate whether SARA-tolerant dairy goats would develop symptoms of SARA or maintain their tolerance under the altered feeding conditions. Sixteen SARA-tolerant goats were selected and fed a high-grain diet (70% concentrate in which crushed corn is the main energy feed) called HGC groups (Additional file 2: Table S8). We subsequently kept feeding dairy goats with diets in Additional file 2: Table S8 for 4 weeks, collecting ruminal fluids to measure the pH values on days 56 and 63 of the trial period and collecting 20 mL blood in the jugular vein with heparin sodium anticoagulation for further VFA concentration measuring on the 63rd day (Additional file 1: Fig S1A). Finally, the individuals with SARA in the high-grain diet group were named HGW-SARA and HGC-SARA, and the individuals without SARA in the high-grain diet group were named HGW-Health and HGC-Health. The diets were prepared as TMR (total mixed rations) and a total of 1 kg TMR experimental diet in dry matter basis was fed to each goat twice daily at 0800 h and 1700 h and all the dairy goats had ad libitum access to water.

#### Cross-transplantation of the rumen microbiome from HGW-SARA and HGC-Health to each other

On the slaughter days, the rumen fluids from three HGW-SARA goats (whose group presented the most severe symptoms of SARA) were transplanted into HGC-Health goats (whose group was the most tolerant of the occurrence of SARA), labelled S + H, whereas the ruminal fluids from three HGC-Health goats were transferred to HGW-SARA goats, designated H + S (Additional file 1: Fig S1B), which were fed the same diets as HGC-Health goats in Additional file 2: Table S8. On the day of the exchange, the rumen contents of the recipient goats were initially evacuated entirely. Subsequently, the rumen was thoroughly rinsed at least three times with 30 l of sterile, prewarmed phosphate-buffered saline (PBS, pH = 6.8) until the rinse solution became colorless. Finally, the rumen contents were transferred. On the 7th and 14th days post-transplantation, we collected ruminal fluids every hour after feeding in the morning to monitored rumen pH for 14 consecutive hours to diagnose the occurrence of SARA.

#### Constructed SARA model of dairy goats and gavaged *Bifidobacterium adolescentis*

To explore the role of *B. adolescentis* in regulating of SARA-susceptibility and tolerance, 14 dairy goats in lactation days (42 ± 5 days) were fed the same high-grain diet with concentrate: forage = 7:3 prepared as TMR, and a total 1 kg TMR in dry matter basis was fed in the morning and afternoon, as detailed in Additional file 2: Table S8. Randomly dividing them into 2 groups, one group was fed 12 mL *B. adolescentis* at 10^8^ CFU/mL named HRDS-BA and another group was fed 12 mL PBS as control called HRDS-C when feeding in the morning and afternoon (Additional file 1: Fig S1C). *B. adolescentis* purchased from the China Center of Industrial Culture Collection (CICC), which was isolated from bovine rumen, was cultivated in Reinforced Clostridium Medium (Additional file 2: Table S9). Liquid medium with *B. adolescentis* was incubated under anaerobic conditions using Anaero Pack systems (Mitsubishi Gas Chemical, Tokyo, Japan) at 37 °C. After 24 h of incubation, 1 mL of the bacterial solution was aspirated to a new RCM medium three times. The third-generation bacterial solution was used for gavage. During the experimental period, rumen fluid was collected via an oral cannula at 0 h, 2 h, 4 h after morning feeding and 2 h, 4 h after afternoon feeding on the 28th and 42nd days. Twenty milliliters of blood was collected from the jugular vein using anticoagulants with heparin sodium at the same time with rumen fluid collection.

#### Sample collection

In LGW-CON, HGW-SARA, HGW-Health, and HGC-Health groups, five dairy goats in each group were euthanized 2 h after feeding in the morning on day 64 and immediately dissected. Before slaughter, 20 mL of blood was collected from the jugular vein and anticoagulated with heparin sodium. The blood samples were centrifuged at 4 °C at 3000 × *g* for 20 min to separate the plasma, divided into 10 parts, and stored at − 20 °C. After slaughter, the rumen fluid was collected and strained through four layers of sterile cheesecloth, stored in liquid nitrogen for 24 h, and then transferred and stored at − 80 °C until further DNA extraction, the non-targeted metabolome determination and rumen fermentation analysis. Then, the rumen epithelial tissue was removed, washed with PBS buffer, and some of them were immediately stored in liquid nitrogen for 24 h, and then transferred and stored at − 80 °C until further RNA extraction and single-nucleus transcriptome sequencing. The other epithelial tissues of the rumen were collected in 2 × 2 cm^2^ pieces and fixed in optimum cutting temperature (OCT) compound to make cryo-embedded tissue. The H + S and S + H dairy goats were slaughtered, and samples were collected on day 14 using the same method as described above.

### Determination of volatile fatty acids (VFAs) in rumen fluid and plasma

The rumen fluid was centrifuged at 13,000 × *g* for 10 min, and then used for VFAs concentration analysis. Meanwhile, the plasma samples were directly used for VFA concentration analysis. We analyzed the concentration of VFAs in rumen fluid and plasma as previously described [5]. In brief, 1 mL ruminal fluid supernatant or plasma was mixed with 200 μL metaphosphoric acid (25% w/v), after 3–4 h of standing at 5 °C, and then centrifuged for 15 min at 13,500 × *g* at 4 °C. Five hundred microliters supernatant was mixed with 200 µL of crotonic acid (28.45 mmol/L) and standing 0.5–2 h at 5 °C, then filtered through a 0.45-µm filter. The VFAs were separated and quantified with an Agilent 7820 A GC system equipped with a polar capillary column (AE-FFAP, 30 m × 0.25 mm × 0.33 μm) and a flame ionization detector (FID).

### Determination of free lipopolysaccharide in both the rumen fluid and plasma

The quantification of free lipopolysaccharides in rumen fluid and plasma from dairy goats was carried out employing the chromogenic endpoint Tachypleus amebocyte lysate assay kit (catalog number EC80545S, Xiamen Houshiji, Ltd.) adhering meticulously to the manufacturer’s guidelines. In summary, rumen fluid samples were subjected to heat treatment at 100 °C for 30 min prior to being stored at −20 °C in preparation for lipopolysaccharide assessment. The assay was performed on a 96-well microplate with the optical density measured at 405 nm on a microplate reader (Model 3550, manufactured by Bio-Rad, Hercules, CA, USA).

### Determination of the lactate and NH_3_-N concentrations in the rumen fluid

The NH_3_-N content of ruminal fluid was determined through the phenol-sodium hypochlorite colorimetric methodology. A standard curve was first established via a gradient series of ammonium standard solutions. In each test tube, 1.0 mL of appropriately diluted sample, standard solution, or distilled water (utilized as a blank control) was dispensed, followed by the addition of 4 mL of HCl solution and thorough mixing. A 0.2-mL aliquot of the mixture was then transferred to separate tubes, whereupon 2.5 mL each of phenol and sodium hypochlorite solutions were sequentially introduced, with subsequent blending. The tubes were subjected to a 10-min color development step in a 60 °C water bath, after which they were quenched in water, and absorbance was measured at a wavelength of 545 nm. Employing the established standard curve, the concentration of NH_4_Cl in individual samples was precisely quantified. The lactic acid content in rumen fluid was assayed using the Rumen Fluid Lactic Acid Kit (A019-2-1, Nanjing Jiancheng Bioengineering Institute), adhering strictly to the kit protocol. Rumen samples, mixed with enzymatic reagents and chromogens, were incubated at 37 °C for 10 min to complete the reaction. Reaction cessation was achieved with a stopping agent, and color intensity, measured at 530 nm with a 1 cm light path, enabled precise colorimetric quantification.

### Morphological observation of the rumen epithelial papilla via H&E staining

Following fixation, the rumen epithelial tissues were subjected to automated dehydration, embedding, sectioning, and subsequent staining with hematoxylin for 10 to 20 min. This was succeeded by a tap water rinse lasting 1 to 3 min, differentiation in hydrochloric acid‒alcohol for 5 to 10 s, and a repeat rinse. To achieve bluening, the sections were bathed in 50 °C warm water or a gently alkaline solution until the appropriate color was obtained. After another 1 to 3 min, the sections were rinsed with tap water, incubated in 85% ethanol for 3 to 5 min and stained with matching amounts of eosin. A swift 3- to 5-s water rinse preceded a graded alcohol dehydration series, xylene clearing, and slide preparation for visualization. Microscopy images of five randomly chosen papillae were then captured at × 100 magnification using a Nikon ECLIPSE Ni-U microscope (Nikon Co. Ltd., Japan). Thereafter, with meticulous attention, the thicknesses of the stratum corneum, granular layer, spinous layer, and overall rumen epithelium within the papillae were measured and documented, contributing vital data to an exhaustive morphometric assessment.

### Immunochemical analysis

Frozen tissue sections were triple rinsed with phosphate-buffered saline (PBS) for 5 min per cycle within a staining chamber and then subsequently submerged in a 5% film dissolving reagent for a 10-min incubation at room temperature, interspersed with another trio of 5-min PBS washes. To suppress nonspecific binding, the sections were uniformly covered with a 10% serum blocking solution and left at ambient temperature for half an hour. Thereafter, the sections were treated with primary antibodies specific for IL17-A, CD163, and KRT6A and incubated overnight at 4 °C. After a trio of 5-min washes with PBS, secondary antibodies were applied, and the samples were allowed to react at 37 °C for 30 min. Nuclear staining was accomplished using DAPI, with a subsequent 10-min incubation. This mixture was capped off with three final 5-min washes in PBS, and finally, the tablets were sealed using anti-fluorescence attenuation tablet sealant. Fluorescence images of the sections were captured using a specialized microscope camera system. Each section was first examined for clear cell visibility, and then three regions were imaged at × 400 magnification. The ImageJ software was used to analyze the images and compute the integrated density (IntDen), area, and mean gray value. The mean fluorescence intensity per sample was derived by averaging the IntDen values from the three images.

### Assays of inflammatory cytokines in the plasma

The concentrations of IL-1β, IL-6, IL-10, TNF-α, and IL-17 in plasma of HRDS-C and HRDS-BA dairy goats were detected using the respective ELISA kits (COIBO BIO, Shanghai, China) with double antibody sandwich method.

### DNA extraction, library construction, and metagenomic sequencing

Total metagenomic DNA was extracted from rumen fluid samples using the E.Z.N.A.® Soil DNA Kit (Omega Bio-tek, Norcross, GA, USA), with repeated bead-beating following the manufacturer’s instructions. Concentration and purity of the extracted DNA were determined with TBS-380 and NanoDrop2000, respectively. The integrity of the DNA was verified through electrophoresis on a 1% agarose gel.

The extracted DNA was cut to an approximate average fragment length of 400 base pairs using the Covaris M220 (Gene Company Limited, China) for paired-end library construction. The NEXTFLEX® Rapid DNA-Seq (Bioo Scientific, Austin, TX, USA) was employed for constructing the paired-end library, wherein adapters, which were generated with sequencing primer hybridization sequences, were ligated to the blunt-ended DNA fragments. Paired-end sequencing was performed on an Illumina NovaSeq 6000 platform (Illumina Inc., San Diego, CA, USA) with NovaSeq Reagent Kits according to the manufacturer’s instructions (www.illumina.com). After bridge PCR amplification, metagenomic sequencing was performed using an Illumina NovaSeq/HiSeq Xten (Illumina, USA) sequencing platform.

### Sequence quality control and genome assembly

Paired-end Illumina reads underwent adaptor trimming and filtering of low-quality sequences—those shorter than 50 bp, possessing a quality score below 20, or containing ambiguous “N” bases—utilizing fastp [69] (version 0.20.0, available at https://github.com/OpenGene/fastp) and Trimmomatic (version 0.39, available at https://github.com/usadellab/Trimmomatic). Reads were aligned to the reference genome of Capra Hircus by BWA [48, 49] (version 0.7.9a, http://bio-bwa.sourceforge.net), and any reads mapped to the genome were discarded to avoid contamination with goat DNA. Metagenomic data assembly was accomplished with MEGAHIT [50] (version 1.1.2, found at https://github.com/voutcn/megahit). Contigs reaching or exceeding 300 bp in length were retained as the final assembly output, which then served as the foundation for downstream gene prediction and annotation. Metagenome sequencing generated a total of 1,785,081,216 reads, with 67,963,658 ± 1,681,281 reads (mean ± SEM) per sample. After quality control and removing host genes, a total of 1,323,991,102 reads were retained, with 67,963,658 ± 1,681,281 (mean ± SEM) per sample.

### Gene prediction, taxonomy, and functional annotation

Open reading frames (ORFs) were inferred from assembled contigs utilizing MetaGene (https://github.com/CharlesJB/metagene). ORFs extending to or beyond 100 base pairs were extracted and translated into amino acid sequences. A nonredundant gene catalog was compiled through the application of CD-HIT [51] (version 4.6.1, https://github.com/weizhongli/cdhit/releases/tag/V4.6.1), which enforces a 90% sequence identity and 90% coverage threshold for clustering. Postquality control reads were mapped to the nonredundant gene catalog with 95% identity via SOAPaligner (version 2.21, https://github.com/ShujiaHuang/SOAPaligner), evaluating quantification of gene abundance within individual samples. The total gene abundance of each sample was normalized to the transcripts per million (TPM) value. Specifically, the gene abundance is expressed as the million-fold proportion of the gene abundance normalized by the gene length, relative to the total abundance of all genes normalized by their lengths in the sample.

For taxonomic classification, representative sequences of the nonredundant gene catalog were aligned to the NCBI NR database using Diamond [52] (version 0.8.35, http://www.diamondsearch.org/index.php), imposing an e-value threshold of 1e − 5. Functional annotations based on the Kyoto Encyclopedia of Genes and Genomes (KEGG) were carried out with Diamond [52] against the KEGG database (http://www.genome.jp/kegg/), maintaining the same e-value cut-off. Carbohydrate-active enzymes (CAZymes) were annotated by subjecting the catalog to hmmscan (http://hmmer.janelia.org/search/hmmscan) analysis against the CAZy database (http://www.cazy.org/).

### Rumen metabolome analysis

Ruminal fluid (1.5 mL) was extracted with methanol:acetonitrile (1:1) and sonicated at 40 kHz for 30 min at 5 °C, followed by protein precipitation at − 20 °C. After centrifugation, the supernatants were lyophilized and then reconstituted in 100 µL of acetonitrile:water (1:1) for UHPLC-MS/MS analysis using the UHPLC-Q Exactive HF-X system (Thermo Fisher Scientific, Massachusetts, USA) after another round of centrifugation and clarification. The LC–MS raw data were imported into the metabolomics processing software Progenesis QI (Waters Corporation, Milford, USA) for baseline filtering, peak identification, integration, retention time correction, and peak alignment. Subsequently, a data matrix of the retention time, mass‒charge ratio, and peak intensity was generated. Metabolite information was obtained by comparing the MS and MS/MS mass spectrum information with the metabolic public database HMDB.

Variance analysis was performed on the matrix file after data preprocessing. The R package ropls (version 1.6.2) was used to perform principal component analysis (PCA) and orthogonal least partial squares discriminant analysis (OPLS-DA), and 7 cycles of interactive validation were used to evaluate the stability of the model. In addition, Wilcox’ test and analysis of fold changes were performed. The selection of significantly differential metabolites was determined based on the variable importance in the projection (VIP) obtained by the OPLS-DA model and the *p* value of Wilcox’ test, and the metabolites with VIP > 1 and *P* < 0.05 were considered significantly different.

Differential metabolites among two groups were summarized and mapped into their biochemical pathways through metabolic enrichment and pathway analysis based on KEGG database. These metabolites can be classified according to the pathways they involved or the functions they performed. Enrichment analysis was usually to analyze a group of metabolites in a function node whether appears or not. The principle was that the annotation analysis of a single metabolite develops into an annotation analysis of a group of metabolites. Scipy.stats (Python package) (https://docs.scipy.org/doc/scipy/) was exploited to identify statistically significantly enriched pathways via Fisher’s exact test.

### Rumen epithelial RNA extraction and sequencing

Total RNA was isolated from rumen epithelial tissue specimens employing TRIzol® Reagent, adhering meticulously to the manufacturer’s guidelines (Invitrogen). Genomic DNA was eliminated through treatment with DNase I (TaKara). The integrity of RNA and the absence of degradation or contaminant bands were verified via 1% agarose gel electrophoresis. Thereafter, the quality of the RNA was assessed using an Agilent 2100 Bioanalyzer (Agilent Technologies) and quantified with a NanoDrop ND-2000 spectrophotometer (NanoDrop Technologies). Solely RNA samples meeting the premium quality criteria (OD260/280 = 1.8 ~ 2.2, OD260/230 ≥ 2.0, RIN ≥ 8.0, 28S:18S ≥ 1.0, > 1 μg) were advanced for the construction of sequencing libraries.

RNA purification, reverse transcription, library construction, and sequencing were executed in accordance with the manufacturer’s protocols (Illumina, San Diego, CA). Transcriptome library preparation was accomplished utilizing the TruSeq™ RNA Sample Preparation Kit from Illumina. Briefly, messenger RNA isolation was achieved through polyA selection employing oligo(dT) beads preceding fragmentation by a dedicated buffer. Thereafter, double-stranded cDNA was generated using a SuperScript Double-Stranded cDNA Synthesis Kit (Invitrogen, CA) in conjunction with random hexamer primers (Illumina). The resulting cDNA underwent end repair, phosphorylation, and adenylation according to Illumina’s recommended library preparation steps. The size of the 300 bp cDNA fragments was selected on a 2% low-range Ultra agarose gel, followed by 15 cycles of PCR amplification with Phusion DNA Polymerase (NEB). Libraries were quantitated by TBS380, and the paired-end RNA-seq library was subsequently sequenced with an Illumina NovaSeq 6000 sequencer (2 × 150 bp read length).

The raw paired-end reads were trimmed and subjected to quality control using fastp [69]. Next, the cleaned reads were mapped to the reference genome utilizing HISAT2 (http://ccb.jhu.edu/software/hisat2/index.shtmL) [70] for orientation alignment. The mapped reads from each sample were assembled with reference guidance by means of StringTie (https://ccb.jhu.edu/software/stringtie/) [71].

To discern differential expression genes (DEGs) between distinct groups, gene expression levels were quantified employing the transcripts per million reads (TPM) method. RSEM (http://deweylab.biostat.wisc.edu/rsem/) [72] was employed for gene expression abundance estimation. Differential expression analysis was primarily conducted with DESeq2 [73], where DEGs with a |log2 (fold change)|≥ 1 and a significance level of *P* value ≤ 0.05 were deemed significantly differentially expressed. Moreover, enrichment analyses for Gene Ontology (GO) terms and KEGG pathways were conducted to pinpoint DEGs showing significant enrichment (*P* value ≤ 0.05). This functional enrichment utilized Goatools (https://github.com/tanghaibao/Goatools) [74] and KOBAS (http://kobas.cbi.pku.edu.cn/home.do) [75] for GO and KEGG analysis.

### Single-nucleus RNA sequencing and analysis

After rinsing with PBS, the rumen epithelial tissue samples stored in liquid nitrogen were subjected to nucleus extraction to prepare single-cell nuclear suspensions. The 10x Genomics ChromiumTM system was employed to generate Gel Bead-in-Emulsion (GEMs), with subsequent GEM collection for reverse transcription and barcoding within a PCR machine. Following lysis of the GEMs, first-strand cDNA was enriched using magnetic beads and purified, followed by cDNA amplification and quality control. Qualifying cDNA underwent next-generation sequencing library preparation, involving fragmentation, adapter ligation, indexing PCR, and final library quantification and quality checks. The paired-end 150 bp (PE150) qualified libraries were subsequently sequenced on the Illumina HiSeq platform.

The quality of the raw read data was analyzed by Fastp [69]. Cell Ranger was used for quality control of the raw data, and the comparison software STAR [76] was used to compare the sequenced reads to the reference genome of Capra Hircus to obtain quality control results, such as the number of high-quality cells, number of genes, and genome comparison information of the sample data. Seruat was used for further quality control filtering and removal of outliers on the basis of initial quality control and standardization in “LogNormalize” [77, 78]. After dimensionality reduction using UMAP, the “SNN” method in Seruat was used for clustering. SingleR was used for cell type identification, and epithelial cell subclusters were identified with reference to the bovine single-cell database of Zhejiang University (http://cattlecelllandscape.zju.edu.cn). All the visualization of UMAP diagrams were based on R packages of Seruat, ggplot2, ggunchull, ggrepel, and tidyverse.

### Quantitative real-time PCR

An appropriate quantity of rumen epithelial tissue was harvested and thoroughly homogenized before lysis. Total RNA extraction was performed utilizing TRIzol reagent (Carlsbad, USA). Thereafter, the extracted RNA was reverse transcribed into cDNA following the standard protocol provided by the PrimeScript® RT Kit (Takara, China). Quantitative real-time polymerase chain reaction (qRT‒PCR) assays were executed with SYBR® Premix Pro TaqTM II (Takara, China) on a real-time fluorescence quantitative thermal cycler (Light Cycler 9,603,030,973). Each 10 μL qRT‒PCR mixture comprised 4 μL of cDNA template, 0.5 μL each of forward and reverse primers, and 5 μL of SYBR Green mix. The PCR cycling conditions entailed an initial denaturation step at 95 °C for 30 s, followed by 40 cycles of 95 °C for 5 s and 60 °C for annealing and extension. The specific primer pairs utilized for the target genes are enumerated in Additional file 2: Table S10. Gene expression levels were analyzed employing the widely accepted approach for relative quantification in qRT‒PCR experiments known as the 2^−ΔΔCt^ method [79].

### Structure equation modelling construction analysis

The structure equation model was constructed in R studio with the R package lavaan [80] to explore the relationships among the rumen microbiome, rumen metabolome, gene expression of the rumen epithelium, and number of epithelial cells. A variance‒covariance matrix was constructed, and the model parameters were estimated utilizing the maximum likelihood method. Prior to commencing the process, the abundance data underwent a transformation to adhere to a normal distribution, specifically adding one to the original value and taking the logarithm. According to the model’s fitting effectiveness, which is referred to as the comparative fit index (CFI), we iterated the model construction several times and compared it with ANOVA. After building successful models, view standardized path coefficients and visualize them.

### Statistical analyses

The power analysis was performed using the R packages "samr" and "limma" on integrated phenotypes alteration, RNA-seq dataset metagenomic datasets and metabolomic datasets to determine the optimal sample size [81]. The results showed that 5 biological replicates per group were sufficient to achieve a statistical power of 0.831, meeting the standard threshold (power > 0.8). The distance algorithm was used to analyze the species and functional composition among samples by PCoA, and the differences between groups were tested by ANOSIM. LEfSe was used to analyze differential microorganisms and their functions, and ggplot2 was used for visualization. DESeq2 software was used to screen DEGs, and KEGG enrichment analysis of DEGs was performed by KOBAS. Differentially abundant metabolites were determined based on the variable weight value (VIP) obtained by the OPLS-DA model and the *P* value of Wilcox’ test, and statistical analysis of differentially abundant metabolites was performed based on Wilcox’ test.

Apart from above, all the statistical analyses were conducted in IBM SPSS Statistics 27 using the mean ± SEM and one-way ANOVA followed by the LSD and Duncan tests. *P < 0.05, **P < 0.01, ***P < 0.001 indicate significance and were visualized with GraphPad Prism 9.5. The correlation analysis was performed in R studio with R packages such as BiocManager, ComplexHeatmap, and circlized with Pearson or Spearman correlation coefficients. ROC curve analysis was also conducted in GraphPad Prism 9.5 with the Wilson/Brown method and 95% confidence intervals.

## Supplementary Information

Additional file 1: Fig S1 The dairy goats experimental design of this research. Fig S2 The differences of rumen fermentation in volatile fatty acids among LGW-CON, HGW-SARA, HGW-Health and HGC-Health. Fig S3 The differences of microbiota species and function between LGW-CON and HGW-SARA. Fig S4 The differences in microbial function between HGW-SARA and HGW-Health goats. Fig S5 There were remarkable differences in rumen epithelial gene expressions between HGW-SARA dairy goats and healthy dairy goats with high RDS (including HGW-Health and HGC-Health). Fig S6 The top 3 cell makers of each subclusters which were identified as epithelial cells. Fig S7 The differences of concentration and proportion in plasma VFAs among SARA and healthy goats. Fig S8 The differences of metabolites among LGW-CON, HGW-SARA, HGW-Health and HGC-Health and differential metabolites identified between HGW-CON and HGW-SARA goats. Fig S9 The differences of rumen microbiota and metabolites among HGW-SARA, HGC-Health, S + H and H + S goats.

Additional file 2: Table S1 the contribution analysis of functions and microbiota. Table S2 The KEGG pathways enriched by differential genes from HGW-SARA vs HGW-Health goats and HGW-SARA vs HGC-Health goats. Table S3 The number of cells of each type in rumen epithelium in each group of dairy goats. Table S4 The quantity and ratio of basal spinous and granule cells. Table S5 The differential compounds in the rumen between HGW-SARA and LGW-CON goats. Table S6 The correlation between microbiome upregulated in HGW-Health and metabolites related with metabolism. TableS7 The differential compounds participated in Tryptophan metabolism in the rumen of HGW-SARA vs H + S and HGW-SARA vs S + H goats. Table S8 Ingredients and nutrient composition of the low and high-concentrate diet on a dry matter (DM) basis. Table S9 The ingredient list of Reinforced Clostridium Medium (RCM). Table S10 The primers used for relative real-time PCR.

## Data Availability

All the data generated or analysed for this study are included in this paper. The metagenome data, RNA-seq data, metabolomics data and single-nucleus RNA-seq data were deposited into the China National Center for Bioinformation (CNCB; https://www.cncb.ac.cn/?lang=en) under accession number PRJCA026627 [82].
