# Supplementary material for: Multi-omics insights into microbiome-rumen epithelium interaction mechanisms underlying subacute rumen acidosis tolerance in dairy goats
Source: Genome Biol. 2025 Oct 9;26:345. doi: 10.1186/s13059-025-03789-y (PMC12509349; doi:10.1186/s13059-025-03789-y)
Supplement: Supplementary file 1 — Additional file 1: Fig S1 The dairy goats experimental design of this research. Fig S2 The differences of rumen fermentation in volatile fatty acids among LGW-CON, HGW-SARA, HGW-Health and HGC-Health. Fig S3 The differences of microbiota species and function between LGW-CON and HGW-SARA. Fig S4 The differences in microbial function between HGW-SARA and HGW-Health goats. Fig S5 There were remarkable differences in rumen epithelial gene expressions between HGW-SARA dairy goats and healthy dairy goats with high RDS (including HGW-Health and HGC-Health). Fig S6 The top 3 cell makers of each subclusters which were identified as epithelial cells. Fig S7 The differences of concentration and proportion in plasma VFAs among SARA and healthy goats. Fig S8 The differences of metabolites among LGW-CON, HGW-SARA, HGW-Health and HGC-Health and differential metabolites identified between HGW-CON and HGW-SARA goats. Fig S9 The differences of rumen microbiota and metabolites among HGW-SARA, HGC-Health, S + H and H + S goats. [file 13059_2025_3789_MOESM1_ESM.pdf]

Multi-omics insights into microbiome-rumen epithelium interaction mechanisms underlying subacute rumen acidosis tolerance in dairy goats

Authors: Jingyi Xu [1,3,5 ※], Xiaodong Chen [1,2,3, ※], Jianrong Ren [1,3, ※], Jiawen Xu [1,3], Lei Zhang [1,3], Fang Yan [1,3], Tao Liu [1,3], Guijie Zhang [2, #], Sharon A. Huws [4, #], Junhu Yao [1, 3, #], Shengru Wu [1, 3,5 #,*]

Author affiliations:

1 College of Animal Science and Technology, Northwest A&F University, Yangling 712100, Shaanxi, China

2 College of Animal Science and Technology, Ningxia University, Yinchuan 750021, China

3 Key Laboratory of Livestock Biology, Northwest A&F University, Yangling 712100, Shaanxi, China

4 Institute of Global Food Security, School of Biological Sciences, Queen's University Belfast, 19 Chlorine Gardens, Belfast, BT9 5DL, Northern Ireland, UK

5 National Center of Technology Innovation for Dairy, Hohhot 010010, Nei Mongol Autonomous Region, China

※These authors contributed equally to the present work and shared the first authorship.

# These authors contributed equally to the present work and shared the co-corresponding authorship: wushengru2013@163.com (SRW), yaojunhu2004@sohu.com (JHY), s.huws@qub.ac.uk (SAH), guijiezhang@nxu.edu.cn (GJZ)

Running title: Susceptibility and tolerance to subacute rumen acidosis

Additional file 1

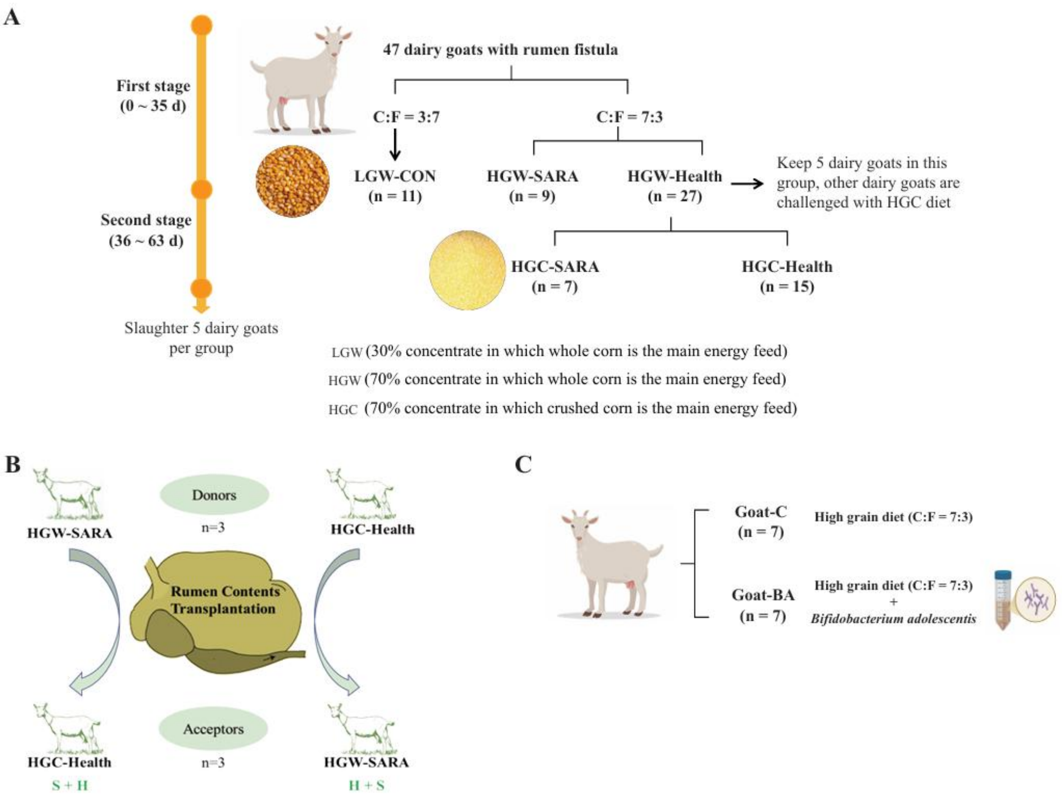

Fig S1 The dairy goats experimental design of this research. **(A)** SARA-susceptibility and tolerance of dairy goats' model construction. **(B)** Cross-transplantation of the rumen microbiome from HGW-SARA and HGC-Health to each other. **(C)** Constructed SARA model of dairy goats and gavaged *Bifidobacterium adolescentis*.

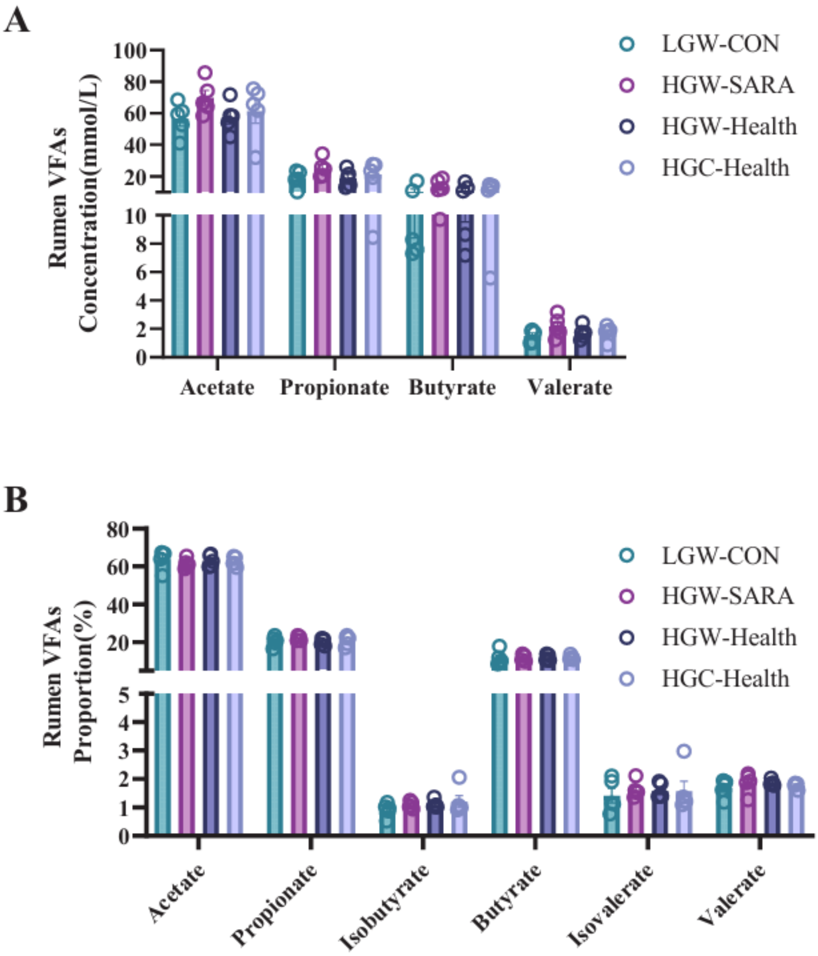

Fig S2 The differences of rumen fermentation in violate fatty acids among LGW-CON, HGW-SARA, HGW-Health and HGC-Health. **(A)** The concentrations of Acetate, Propionate, Butyrate and Valerate in rumen fluids among LGW-CON, HGW-SARA, HGW-Health and HGC-Health. **(B)** The proportions of Acetate, Propionate, Isobutyrate, Butyrate, Isovalerate and valerate in rumen fluids among LGW-CON, HGW-SARA, HGW-Health and HGC-Health.

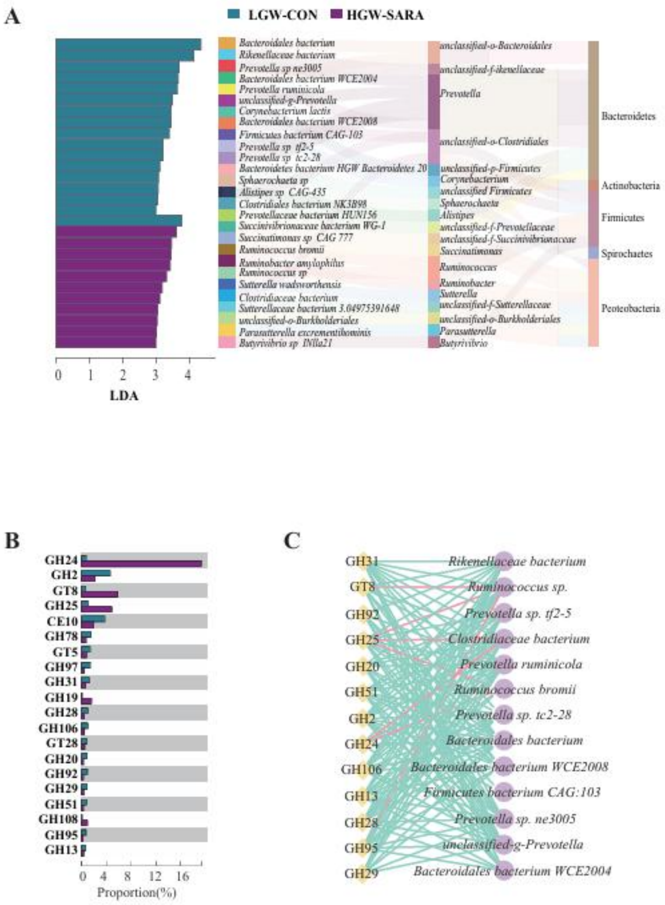

Fig S3 The differences of microbiota species and function between LGW-CON and HGW-SARA. **(A)** LEfSe analysis and Sankey diagram showing differential rumen bacteria between LGW-CON and HGW-SARA goats at the phylum to species level (LDA > 2, $P < 0.05$). **(B)** The differential CAZy enzymes between LGW-CON and HGW-SARA goats (LDA > 2, $P < 0.05$) according to LEfSe analysis. **(C)** The top 20 most abundant CAZy enzymes and the top 30 most abundant bacteria were screened, and the contribution of bacteria to function was calculated. A comparison of the difference in the degree of contribution between the two groups revealed that the degree of contribution was greater in the HGW-SARA group (pink), and the degree of contribution was greater in the LGW-CON group (green).

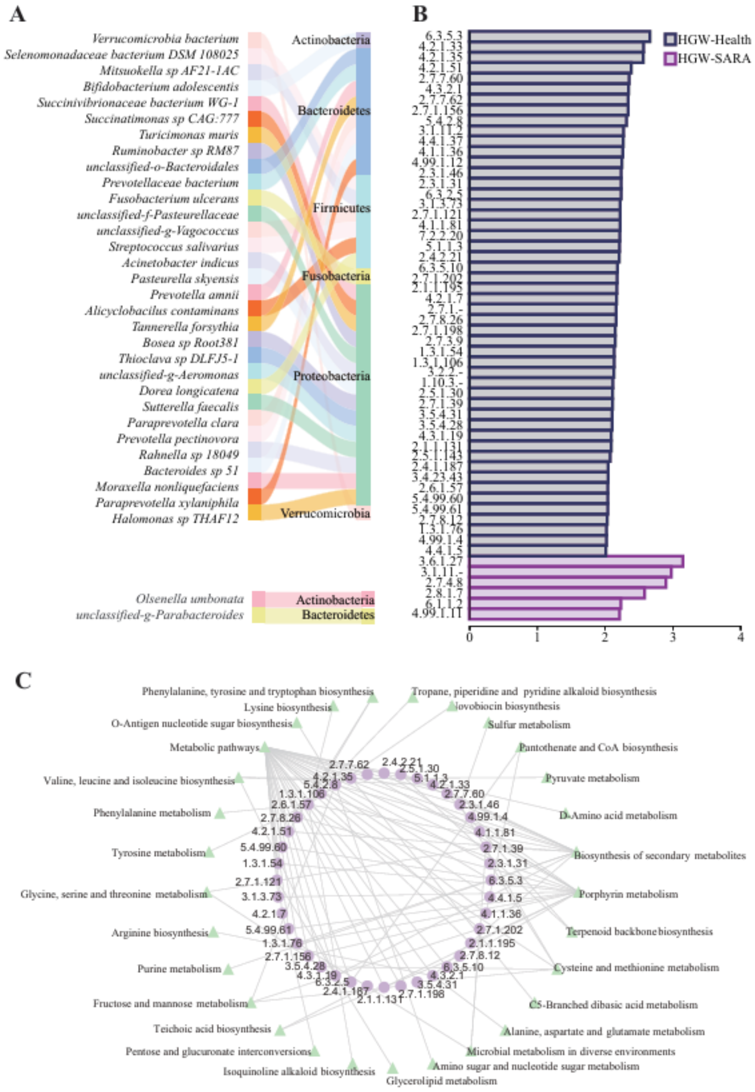

Fig S4 The differences in microbial function between HGW-SARA and HGW-Health goats. **(A)** The Sankey plot to demonstrate the subordinate relationship between species and phylum. **(B)** Lefse analysis was used to investigate the differences of KEGG enzyme between the HGW-SARA and HGW-Health goats (LDA > 2, $P < 0.05$). **(C)** Network diagram showed the KEGG enzymes involved in the key KEGG pathways with differences between the two groups of HGW-SARA and HGW-Health.

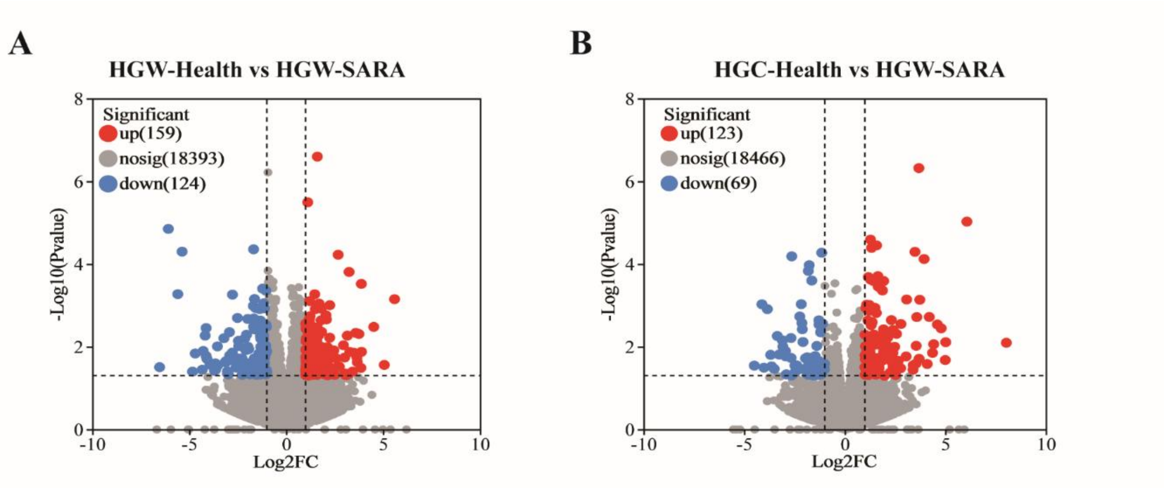

Fig S5 There were remarkable differences in rumen epithelial gene expressions between HGW-SARA dairy goats and healthy dairy goats with high RDS (including HGW-Health and HGC-Health). **(A)** Volcano plots showed differentially expressed genes between HGW-SARA and HGW-Health, where red is significantly up-regulated in the HGW-Health goats and blue is significantly down-regulated in the HGW-Health goats (|log2FC| ⩾ 1, P < 0.05). **(B)** Volcano plots showed differentially expressed genes between HGW-SARA and HGC-Health, where red is significantly up-regulated in the HGC-Health goats and blue is significantly down-regulated in the HGC-Health goats (|log2FC| ⩾ 1, P < 0.05).

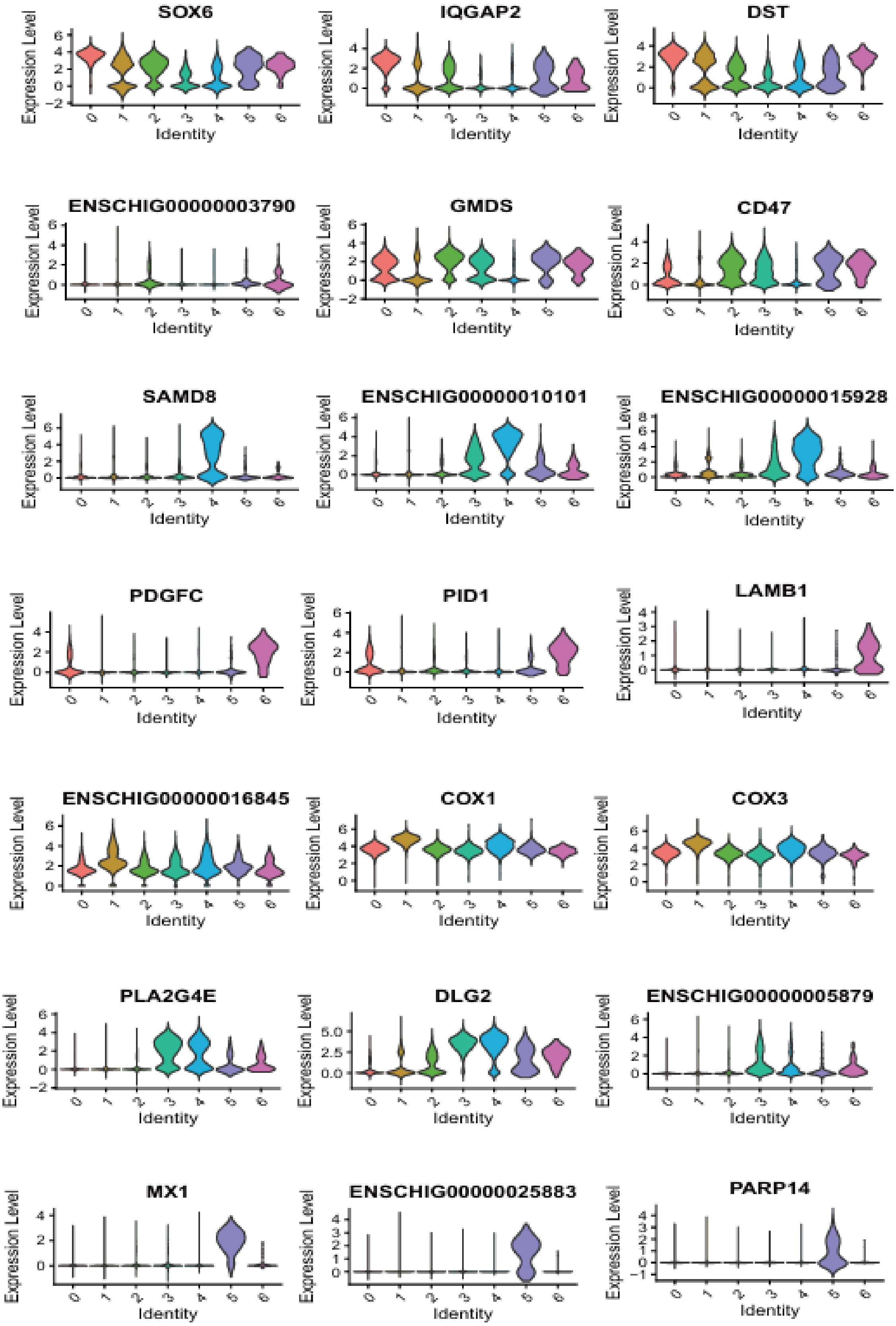

Fig S6 The top 3 cell makers of each subclusters which were identified as epithelial cells.

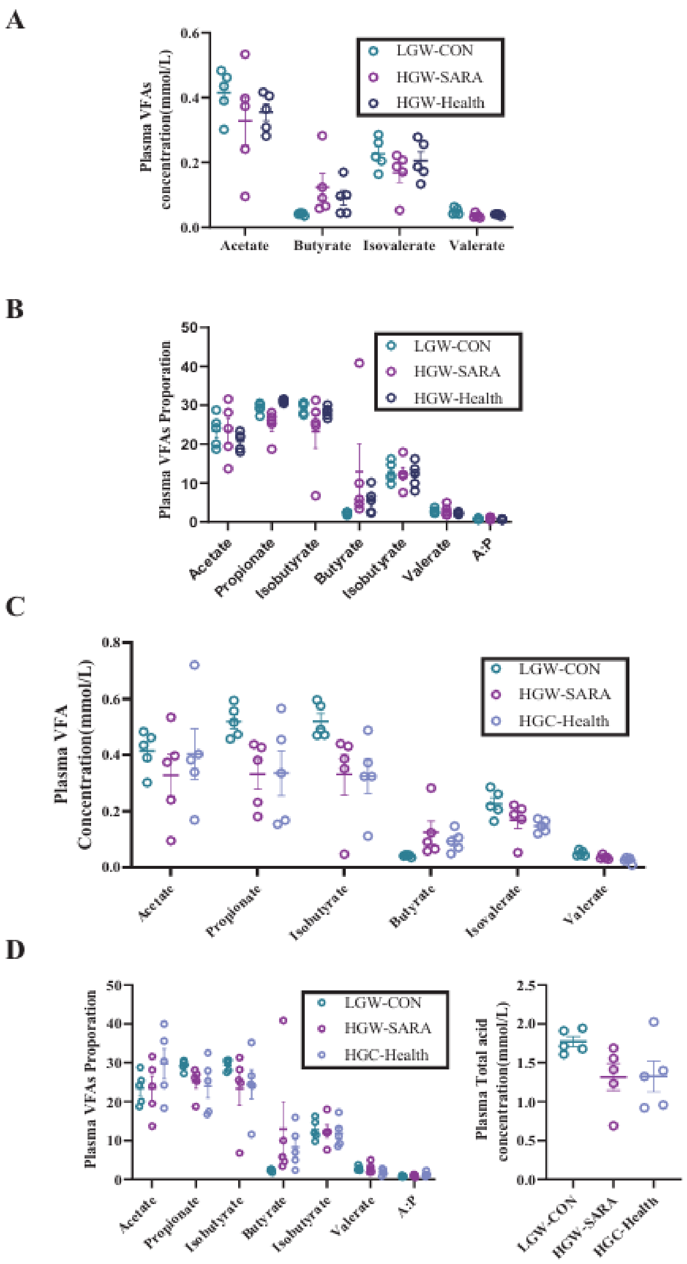

Fig S7 The differences of concentration and proportion in plasma VFAs among SARA and healthy goats. **(A)** The comparison of concentrations of Acetate, Butyrate, Isovalerate and Valerate in serum among LGW-CON, HGW-SARA, HGW-Health. **(B)** The comparison of proportion of Acetate, Propionate, Isobutyrate, Butyrate, Isobutyrate, Valerate and the ratio of Acetate to Propionate among LGW-CON, HGW-SARA and HGW-Health. **(C)** The comparison of concentrations of Acetate, Propionate, Isobutyrate, Butyrate, Isovalerate and Valerate in serum among LGW-CON, HGW-SARA, HGC-Health. **(D)** The comparison of proportion of Acetate, Propionate, Isobutyrate, Butyrate, Isobutyrate, Valerate and the ratio of Acetate to Propionate, as well as the total acids in serum among LGW-CON, HGW-SARA and HGC-Health.

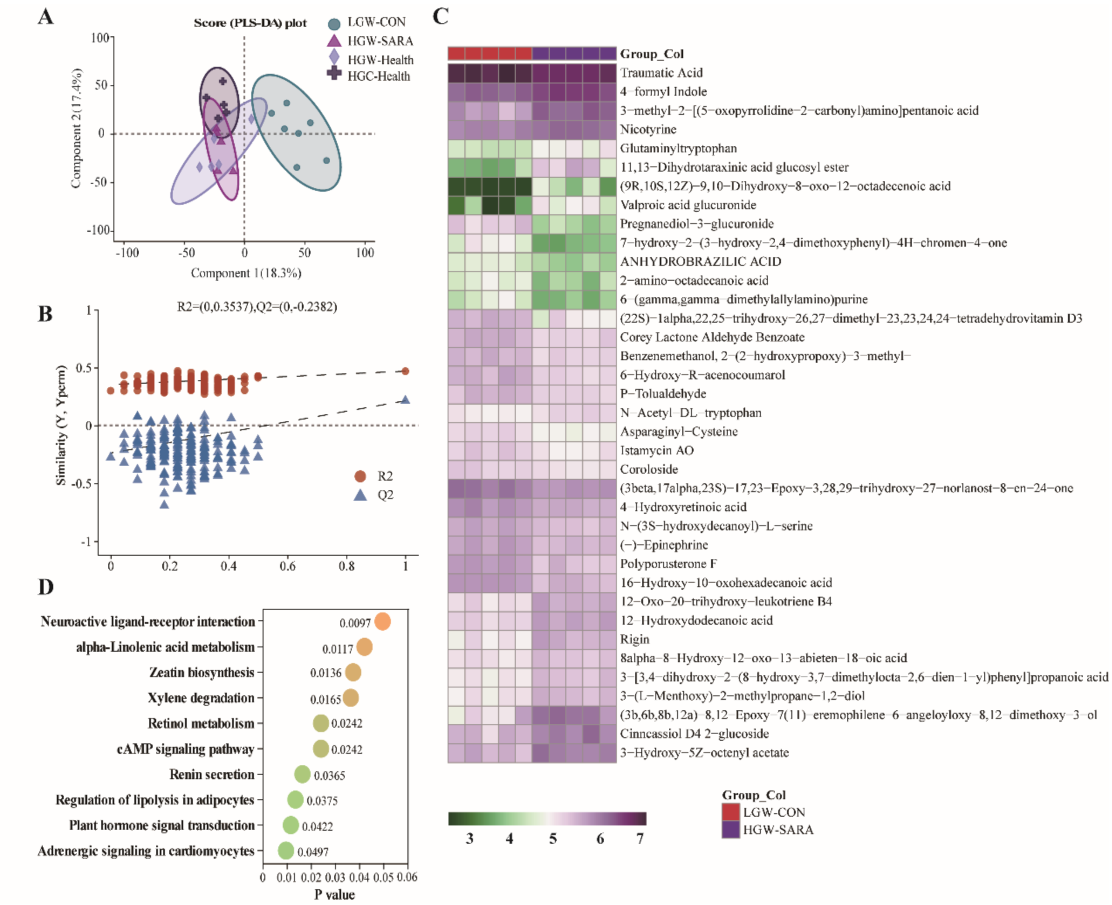

Fig S8 The differences of metabolites among LGW-CON, HGW-SARA, HGW-Health and HGC-Health and differential metabolites identified between HGW-CON and HGW-SARA goats. **(A)** Partial least squares discriminant analysis (PLS-DA) showed the clustering and differences in metabolites among LGW-CON, HGW-SARA, HGW-Health and HGC-Health. **(B)** PLS-DA permutation test, the number of permutation is 200 times, the vertical coordinate represents the value of R2 (blue dot) and Q2 (red triangle) permutation test, and the two dashed lines represent the regression line of R2 and Q2 respectively. Q2 intercept between regression line and Y-axis. Intercept less than 0.05 indicates that the model is robust and reliable, and no overfitting occurs. **(C)** The heatmap showed the differential metabolites between LGW-CON and HGW-SARA ($P < 0.05$, VIP > 1). **(D)** The KEGG enrichment analysis showed the significantly differential KEGG pathways between LGW-CON and HGW-SARA ($P < 0.05$).

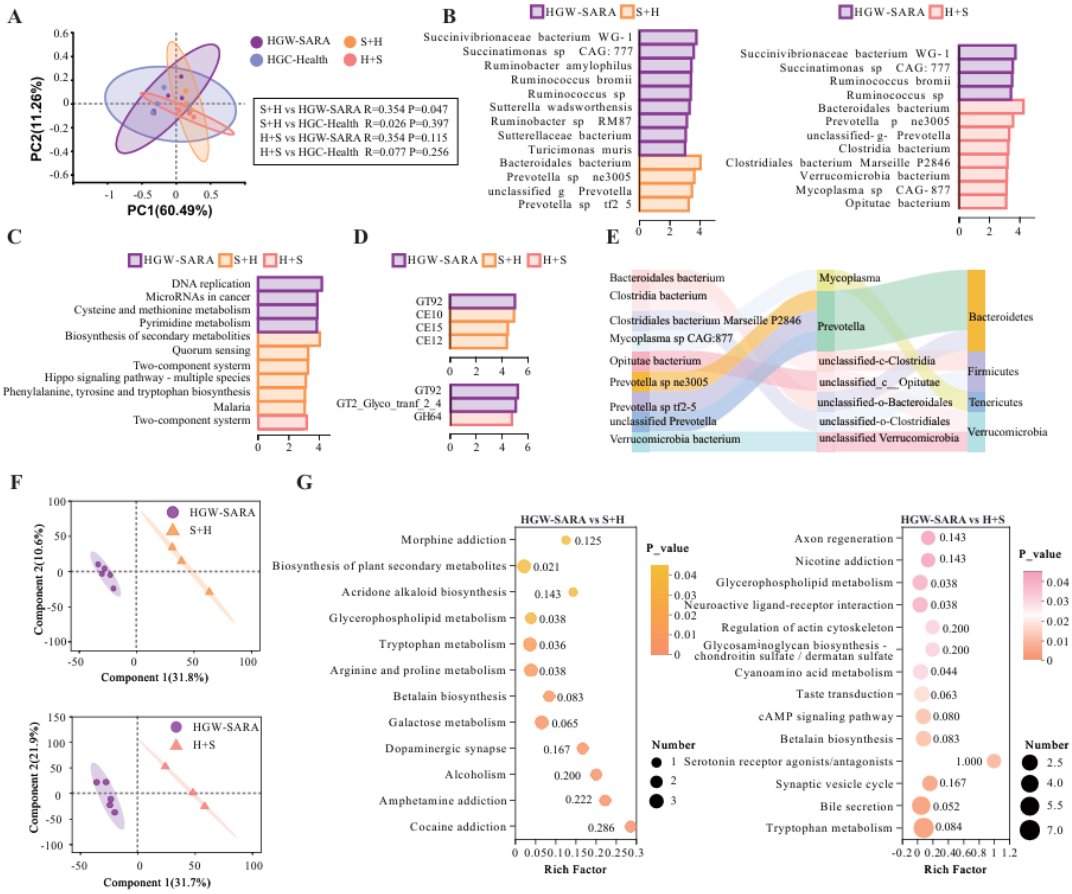

Fig S9 The differences of rumen microbiota and metabolites among HGW-SARA, HGC-Health, S+H and H+S goats. **(A)** The β diversity in PcoA of rumen microbe in HGW-SARA, HGC-Health, S+H and H+S. **(B)** Lefse analysis showed differential rumen bacterial in HGW-SARA vs S+H and HGW-SARA vs H+S goats (LDA > 3, $P < 0.05$). **(C)** Lefse analysis showed differential functions in KEGG pathway level 3 of rumen bacterial in HGW-SARA vs S+H and HGW-SARA vs H+S goats (LDA > 3, $P < 0.05$). **(D)** Lefse analysis showed differential CAZy enzymes of rumen bacterial in HGW-SARA vs S+H and HGW-SARA vs H+S goats (LDA > 2, $P < 0.05$). **(E)** The Sankey map showed genus-level and phylum-level classification of microbiota enriched in H+S and S+H goats compared to HGW-SARA. **(F)** Partial least square discriminant analysis (PLS-DA) compared the clustering and difference of HGW-SARA and S+H samples showing on the top and HGW-SARA and H+S samples showing on the bottom. **(G)** The KEGG pathways significantly enriched by differential metabolites between HGW-SARA and S+H on the left, and between HGW-SARA and H+S on the right.
